# Early-life stress and dietary fatty acids impact the brain lipid/oxylipin profile into adulthood, basally and in response to LPS

**DOI:** 10.3389/fimmu.2022.967437

**Published:** 2022-09-05

**Authors:** Kitty Reemst, Jelle Y. Broos, Maralinde R. Abbink, Chiara Cimetti, Martin Giera, Gijs Kooij, Aniko Korosi

**Affiliations:** ^1^ Swammerdam Institute for Life Sciences, Center for Neuroscience, University of Amsterdam, Science Park, Amsterdam, Netherlands; ^2^ Amsterdam University Medical Center (UMC), Vrije Universiteit Amsterdam, Department of Molecular Cell Biology and Immunology, Amsterdam Neuroscience, Multiple Sclerosis (MS) Center Amsterdam, Amsterdam, Netherlands; ^3^ Center for Proteomics and Metabolomics, Leiden University Medical Center, Leiden, Netherlands

**Keywords:** early-life stress, LPS, lipidomics, Oxylipin, PUFA, dietary intervention

## Abstract

Brain lipid dysregulation is a hallmark of depression and Alzheimer’s disease, also marked by chronic inflammation. Early-life stress (ELS) and dietary intake of polyunsaturated fatty acids (PUFAs) are risk factors for these pathologies and are known to impact inflammatory processes. However, if these early-life factors alter brain lipid homeostasis on the long-term and thereby contribute to this risk remains to be elucidated. We have recently shown that an early diet enriched in omega(ω)-3 PUFAs protected against the long-term negative effects of ELS on cognition and neuroinflammation. Here, we aim to understand if modulation of brain lipid and oxylipin profiles contributes to the detrimental effects of ELS and the protective ones of the diet. We therefore studied if and how ELS and early dietary PUFAs modulate the brain lipid and oxylipin profile, basally as well as in response to an inflammatory challenge, to unmask possible latent effects. Male mice were exposed to ELS *via* the limited bedding and nesting paradigm, received an early diet with high or low ω6/ω3 ratio (HRD and LRD) and were injected with saline or lipopolysaccharide (LPS) in adulthood. Twenty-four hours later plasma cytokines (Multiplex) and hypothalamic lipids and oxylipins (liquid chromatography tandem mass spectrometry) were measured. ELS exacerbated the LPS-induced increase in IL-6, CXCL1 and CCL2. Both ELS and diet affected the lipid/oxylipin profile long-term. For example, ELS increased diacylglycerol and LRD reduced triacylglycerol, free fatty acids and ceramides. Importantly, the ELS-induced alterations were strongly influenced by the early diet. For example, the ELS-induced decrease in eicosapentaenoic acid was reversed when fed LRD. Similarly, the majority of the LPS-induced alterations were distinct for control and ELS exposed mice and unique for mice fed with LRD or HRD. LPS decreased ceramides and lysophosphotidylcholine, increased hexosylceramides and prostaglandin E_2_, reduced triacylglycerol species and ω6-derived oxylipins only in mice fed LRD and ELS reduced the LPS-induced increase in phosphatidylcholine. These data give further insights into the alterations in brain lipids and oxylipins that might contribute to the detrimental effects of ELS, to the protective ones of LRD and the possible early-origin of brain lipid dyshomeostasis characterizing ELS-related psychopathologies.

## 1 Introduction

There is increasing evidence that lipid dysregulation in the brain might represent a key event in the pathophysiology of neurological diseases such as depression and Alzheimer’s disease (1–7). Importantly, early-life stress (ELS) and dietary fatty acids (FA), have been shown to greatly contribute to the risk of developing such psychopathologies (8–19) and Alzheimer’s disease (20–27). In particular, the interaction of such early-life elements is of great interest as we have recently shown that early diet enriched with omega(ω)-3 polyunsaturated fatty acids (PUFA’s) can protect against the long-term negative effects of ELS on cognition and neuroinflammation (28). It is thus intriguing to hypothesize that lipid dysregulation associated with these disorders might have an early-life origin, and to further our insights about if and how alterations in brain lipid composition might contribute to the ELS-induced effects as well as to the protective effects of the early diet enriched with ω3 PUFAs.

It is undoubted that brain lipids are essential for brain function. In fact the brain, apart from the adipose tissue, is the most lipid rich organ of mammals, with approximately 75% of all lipids being exclusive to neural tissues and essential for its structure and function (e.g. energy storage, formation of cellular membranes, cell signaling and regulation of (neuro)inflammation (29–31). Lipids are classified into classes and species, each performing specific biological functions (32–35). For example, sphingolipids, such as ceramides and sphingomyelin and their metabolites play an important role in maintaining membrane integrity and function as signaling molecules for regulating cell proliferation, differentiation, survival and apoptosis (26, 36). PUFA’s are especially enriched in the brain and are implicated in brain development, neuroplasticity and neuroinflammation (30, 37). PUFAs are essential nutrients, as the majority needs to be taken up *via* the diet after which they are transferred to the brain *via* the blood as free fatty acids bound to albumin (38, 39). During inflammatory conditions PUFAs are converted into pro- and anti-inflammatory oxylipins involved in both the promotion and resolution of inflammation (30, 40–43). Impaired resolution of inflammation might be one of the propagating factors for chronic inflammation which is characteristic for depression and Alzheimer’s disease (44–48).

There is emerging evidence from human and animal studies that ELS might indeed impact on lipid metabolism (28, 49–51), which has mostly been addressed in the blood. For example ELS exposure has been associated with reduced PUFAs in plasma of low-income children (49) and resulted in an altered lipidomic profile in serum of major depressive disorder patients (50). There is recent indication that such lipid changes might hold true also within the brain, as postmortem brains of depressed suicides with a history of child abuse exhibited fatty acid dysregulation in the anterior cingulate (51). It is important to note that in these studies dietary intake was not assessed, and thus it remains unclear to what extent potential differences in dietary (FA) intake might contribute to the observed altered plasma and serum lipid levels and brain fatty acid dysregulation. In line with the human findings, we have previously demonstrated in a chronic ELS mouse model that, ELS leads to short- and long-term changes in FA status in peripheral and central tissues (28). Notably, in these animal studies dietary (FA) intake was controlled for suggesting that next to potential dietary influences, other elements of the ELS exposure contribute to lipid and FA status.

Similarly, dietary FA manipulations have been shown to be able to modulate brain lipid composition. For example, we demonstrated that lowering the ratio of ω6 to ω3 PUFAs (15:1 versus 1:1) fed early in life (from postnatal day (P2) until P42), was able to restore ELS-induced changes in FA composition at P9 and, as mentioned above protected against ELS-induced long-term cognitive deficits and alterations in brain plasticity (hippocampal neurogenesis and neuroinflammation) in adulthood (28). Notably, the ratio between dietary ω6 linoleic acid (LA) and ω3 a-linolenic acid (ALA) is a key determinant, of ω3 PUFA status because LA and ALA compete for conversion to their respective PUFAs by the same enzymes. The current LA/ALA ratios were specifically chosen, as also in our earlier study (28), to mimic the shift toward an increased intake of dietary LA in our modern society with the high ratio (15:1)  (52) which has been associated with psychopathologies, while the low LA/ALA (1:1) ratio has been shown to optimize PUFA status (53–59).

In addition, dietary FA manipulations also affect the expression of oxylipin synthesizing enzymes as well as oxylipin levels, both in peripheral tissues and in the CNS (60–63). For instance, Rey and colleagues reported that a two-month dietary ω3 PUFA supplementation (as compared to mice fed a diet deficient in ω3 PUFAs) increased hippocampal ω3 PUFA levels and related downstream oxylipin-derivatives and decreased ω6 derived oxylipins when measured directly after completion of the dietary intervention, both under basal conditions as in response to an inflammatory lipopolysaccharides (LPS) challenge (63). This data suggests that dietary PUFA’s might be able to promote the resolution of neuroinflammation through the release of oxylipins.

Currently, if and how ELS and early dietary ω6/ω3 ratio affect the brain lipidome and related oxylipin levels during development into adulthood is unknown. In addition, it is unclear whether early-dietary ω6/ω3 ratio induced changes in lipid profile might contribute to the protective effect of the diet in the context of ELS induced learning impairments (28). In order to study the long-lasting impacts of early-life exposures, it is often necessary to study these changes not only at basal level but also in response to a ‘second hit’ in order to unmask possible latent effects of these early-life exposures (64, 65). Consequentially, we examined the effects of ELS and early dietary high versus low ω6/ω3 ratio, both under basal conditions as well as in response to an LPS challenge in adulthood, on i) plasma cytokines, ii) hypothalamic lipids and oxylipin composition and iv) correlations thereof.

We observed that i) ELS and diet impact brain lipid and oxylipin profiles long-term, both basally as well as in response to LPS, ii) the ELS-induced effects are highly dependent on the early diet and iii) similarly, the LPS-induced changes are dependent on both ELS and early diet exposure. This data suggests that lipid dysregulation might have an early-life origin and that it potentially contributes to the ELS and diet mediated risk for psychopathologies and cognitive decline.

## 2 Material and methods

### 2.1 Animals

All mice (C57BL/6J) were kept under standard housing conditions with a temperature between 20 and 22°C, a 40 to 60% humidity level, and provided with chow and water ad libitum. Mice were kept on a standard 12/12h light/dark schedule (lights on at 8AM). All experimental procedures were conducted under national law and European Union directives on animal experiments, and were approved by the animal welfare body of the University of Amsterdam.

**Figure 1:**
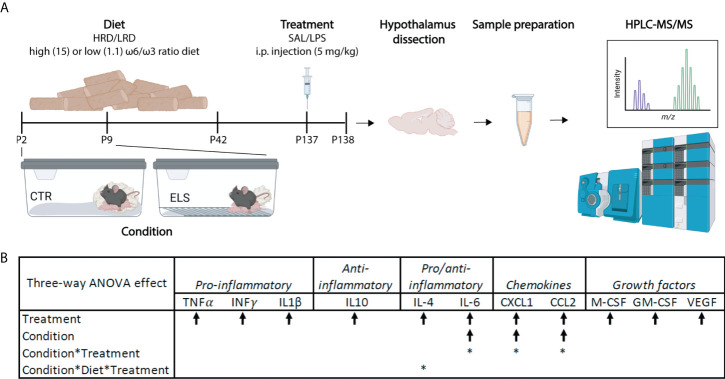
Early-life stress exacerbates LPS induced levels of plasma IL6, CXCL1 and CCL2. **(A)** Experimental timeline. Created with Biorender.com **(B)** Significant three-way ANOVA effects on plasma cytokine levels; Condition (CTL/ELS); Treatment (SAL/LPS); Diet (HRD/LRD). Arrow indicates the direction of the main effect, *: significant interaction effect, p < 0.05. ANOVA, analysis of variance; CTL, control; ELS, early-life stress; SAL, saline; LPS, lipopolysaccharide; HRD, high ω6/ω3 ratio diet; LRD, low ω6/ω3 ratio diet; HPLC-MS/MS, high performance liquid chromatography-tandem mass spectrometry.

Briefly, male mice were exposed to ELS *via* limited bedding and nesting (LBN) paradigm (P2 to P9) (section 2.3) and to an early diet (P2 – P42) with either high (15:1) or low (1.1:1) ω6 linoleic acid to ω3 alpha-linolenic acid ratio (section 2.4). In adulthood mice were injected with either saline or LPS (section 2.5). Hypothalamic lipid and oxylipins were analyzed *via* high performance liquid chromatography-tandem mass spectrometry (HPLC-MS/MS; section 2.8, 2.9 and 2.10; **Figure 1A**). Control mice fed a diet with high ω-6/ω-3 ratio and injected with saline: *CTL-HRD-SAL*; ELS exposed mice fed high ω-6/ω-3 ratio and injected with saline: *ELS-HRD-SAL*; control mice fed a diet with low ω-6/ω-3 ratio and injected with saline: *CTL-LRD-SAL* and ELS mice fed a diet with low ω-6/ω-3 ratio and injected with saline: ELS-LRD-SAL; control mice fed a diet with high ω-6/ω-3 ratio and injected with LPS: *CTL-HRD-LPS*; ELS exposed mice fed high ω-6/ω-3 ratio and injected with LPS: *ELS-HRD-LPS*; control mice fed a diet with low ω-6/ω-3 ratio and injected with LPS: *CTL-LRD-LPS* and ELS mice fed a diet with low ω-6/ω-3 ratio and injected with LPS: *ELS-LRD-LPS*. See **Table 1** for sample size per experimental group per dataset. For clarity, all our predictor variables are referred to in *italics* to distinguish them from the many abbreviations used in this manuscript for lipid classes, species and oxylipins.

**Table 1:** Experimental groups and sample size per dataset.

Condition	CTL				ELS			
Diet	HRD		LRD		HRD		LRD	
Treatment	SAL	LPS	SAL	LPS	SAL	LPS	SAL	LPS
Broad lipidomic dataset	CTL-HRD-SAL (8)	CTL-HRD-LPS (6)	CTL-LRD-SAL (7)	CTL-LRD-LPS (8)	ELS-HRD-SAL (7)	ELS-HRD-LPS (8)	ELS-LRD-SAL (7)	ELS-LRD-LPS (8)
Oxylipin dataset	CTL-HRD-SAL (8)	CTL-HRD-LPS (7)	CTL-LRD-SAL (8)	CTL-LRD-LPS (8)	ELS-HRD-SAL (7)	ELS-HRD-LPS (9)	ELS-LRD-SAL (7)	ELS-LRD-LPS (8)
Cytokine dataset	CTL-HRD-SAL (11)	CTL-HRD-LPS (7)	CTL-LRD-SAL (12)	CTL-LRD-LPS (9)	ELS-HRD-SAL (12)	ELS-HRD-LPS (7)	ELS-LRD-SAL (11)	ELS-LRD-LPS (8)

CTL, control; ELS, early-life stress; SAL, saline; LPS, lipopolysaccharide; HRD, high ω6/ω3 ratio diet; LRD, low ω6/ω3 ratio diet.

### 2.2 Breeding

Experimental mice were bred in house to standardize the perinatal environment. 10-week-old female and 8-week-old male mice were purchased from Harlan Laboratories B.V. (Venray, The Netherlands) and habituated for two weeks before onset of breeding. After the habituation period, two females and one male were housed together for one week to allow mating. Breeding males were removed after one week and after another week of paired-housing, pregnant primiparous females were individually housed in a standard cage with a filtertop. To ensure a stable, quiet environment, cages were placed in a ventilated, airflow-controlled cabinet. Birth of pups was monitored every 24 hours. Litters born before 9:00 AM were considered postnatal day (P)0 on the previous day.

### 2.3 Early-life stress paradigm

The early-life stress (ELS) paradigm consisted of limiting the nesting and bedding material from P2 to P9 as previously described (28, 66–68). On the morning of P2, dams and pups were randomly assigned to the control (CTL) or ELS condition. Litters were culled to six pups with a minimum of 5 pups to prevent maternal care variation due to variable litter size. Litters included at least one male and one female. At P2, dams and pups were weighed and housed under CTL or ELS conditions. CTL cages contained standard amounts of sawdust bedding and one square, cotton piece of nesting material (5x5 cm; Technilab-BMI, Someren, The Netherlands). ELS cages contained fewer amounts of sawdust bedding, only covering the bottom of the cage, a fine-gauge stainless steel mesh raised 1 cm above the cage floor, and half a square cotton piece of nesting material (2,5x5 cm). Cages were covered with a filtertop and placed in a ventilated, airflow-controlled cabinet to ensure a stable, quiet environment and reduce external stressors. Throughout all procedures, manipulation was kept to a minimum to avoid handling effects and animals were left undisturbed until P9. At P9, pups were weighed and moved to standard cages. Mice were weaned at P21 and housed with same-sex littermates in groups of 2 to 3 mice per cage. Only male offspring was used for experimental procedures.

### 2.4 Dietary intervention

Dams were assigned to the American Institute of Nutrition-93 (AIN-93G/M) semi-synthetic diet throughout the breeding period (69). Experimental diets were provided from P2 onwards to dam with litter, and after weaning (P21) offspring were kept on their respective diet until P42. The two experimental diets (Ssniff-Spezialdiäten GmbH, Soest, Germany) were semi-synthetic differing only in LA/ALA ratio that was either a high (15:1) or low (1.1:1). For exact composition of the diets see **Table 2**. The diets were isocaloric and contained a macro- and micronutrient composition according to the AIN93-G purified diets for laboratory rodents (69). Following dietary intervention at P42, all mice were fed AIN-93M until the end of the experiment.

**Table 2:** Composition of experimental high and low ω6/ω3 PUFA diets (grams/kilogram diet).

Ingredient	High ω6/ω3 (15)	Low ω6/ω3 (1.1)
Cornstarch, pregelatinized	397.5	397.5
Casein	200.0	200.0
Maltodextrin 10 DE	132.0	132.0
Sucrose	100.0	100.0
Cellulose	50.0	50.0
Mineral premix	35.0	35.0
Vitamin premix	10.0	10.0
L-cystein	3.0	3.0
Choline CL (50%)	2.5	2.5
Oil blend	70.0	70.0
Coconut oil. hydrogenated	23.8	23.6
Peanut oil	21.3	20.0
Safflower oil	20.2	6.7
Linseed oil	2.2	19.7
Soybean oil	2.5	_
Fatty acids (% total fatty acids)		
C6:0	0.1	0.1
C8:0	1.9	1.9
C10:0	1.9	1.9
C12:0	15.3	15.4
C14:0	6.9	6.9
C16:0	9.6	9.1
C18:0	6.0	6.3
C20:0	1.0	0.9
∑ SFA	42.6	42.4
C18:1	21.6	22.1
C20:1	0.6	0.4
∑ MUFA	22.1	22.6
C18:2n-6 (LA)	30.6	17.3
C18:3n-3 (ALA)	2.0	15.3
∑ PUFA	32.6	32.6
LA/ALA	15.3	1.1

PUFA, polyunsaturated fatty acids; MUFA, monounsaturated fatty acids; LA, linoleic acid; ALA, α-linolenic acid; SFA, short chain fatty acids.

### 2.5 Lipopolysaccharide injection

In adulthood (P137) mice received an intraperitoneal (i.p.) injection of sterile saline (SAL) or 5 mg/kg lipopolysaccharide (LPS, strain O111:B4, Sigma-Aldrich) dissolved in sterile saline. 24 hours following the injection, mice were weighed and sacrificed.

### 2.6 Tissue collection and plasma corticosterone and cytokine measurements

Mice were sacrificed *via* rapid decapitation. Trunk blood was collected and hypothalami were quickly dissected and snap-frozen on dry ice. Tissue was stored at -80°C (brain) or -40°C (blood) until further processing. Plasma from trunk blood was used to measure corticosterone (CORT) levels and cytokine profiles. CORT was measured using a commercially available radioimmunoassay kit (MP Biomedicals, Eindhoven, The Netherlands) according to the manufacturer’s instructions. Plasma levels of pro- and anti-inflammatory cytokines, chemokines and growth factors (TNFα, IFNγ, IL1β, IL10, IL4, IL6, CXCL1, CCL2, M-CSF, GM-CSF, VEGF) were assessed using Milliplex (mouse cytokine/chemokine magnetic bead panel, Milliplex, Merck) according to the manufacturer’s instructions.

### 2.7 Statistical analyses for body weight, food intake, plasma corticosterone and cytokine measurements

Data were analyzed using SPSS 20.0 (IBM software), Graphpad Prism 5 (Graphpad software), and R statistical software (R 3.4.1, http://cran.r-project.org/). Data were expressed as mean ± standard error of the mean (SEM) and considered statistically significant when p<0.05. BW gain and food intake during early-life was analyzed per litter. Data with two predictor variables (condition (*CTL/ELS*) and diet (*HRD/LRD*)) were analyzed using two-way-analysis of variance (ANOVA) and data with three predictor variables (condition (*CTL/ELS*), diet (*HRD/LRD*), and treatment (*SAL/LPS*)) were analyzed using three-way-ANOVA. As multiple mice from a litter were included in experiments, litter corrections were performed when a significant contribution of litter was found in a mixed model analysis with litter included as random factor.

### 2.8 Broad lipidomic analysis

The Lipidyzer™ Platform (SCIEX, Framingham, USA) was used to perform quantitative lipidomics of the collected hypothalami samples as described previously in (70). Briefly, the hypothalami were weighted and homogenized in 2-propanol to a final concentration of 30mg/ml by bullet blending. 75uL of this extract was used for broad lipidomics and 200uL of this extract was used for the oxylipin analysis (described in Method section 2.9). A mix of deuterated internal standards (IS; SCIEX cat# 504156) was added to the broad lipidomic extract after which the lipids were extracted by methyl-tert-butyl ether extraction. The combined organic extracts were dried under a gentle stream of nitrogen and reconstituted with Lipidyzer running buffer. Subsequently, acquisition and quantification were performed using the Lipidyzer™ platform, consisting of a QTrap 5500 mass spectrometer (SCIEX) with differential mobility separation device (DMS), coupled to a Shimadzu Nexera X2 LC system, for flow injection, and the Lipidomics workflow manager software. Internal calibration was used to quantify the lipid species. For the internal calibration, deuterated IS lipids for each lipid class were used within the lipidomics workflow manager. Briefly, each lipid species was corrected by the closest deuterated IS within its lipid class and afterwards the obtained area ratio was multiplied by the concentration of the IS and further corrected for the volume and weight of the sample. The original Lipidyzer platform employed in the present study was not strictly adhering to current LIPID MAPS shorthand notation rules. Nevertheless, for simplified data handling we used the output format of the platform. In order to clarify this as well as the level of identification detail for the various lipids reported, please see **Supplementary Material 1**, which correlates the Lipidyzer output with the current LIPID MAPS shorthand notation system. For additional translation to the lipid shorthand annotation and a detailed description of the Lipidyzer platform see (70).

### 2.9 Oxylipin analysis

The measurement of oxylipin and their precursors (further referred to as “oxylipin dataset”) was performed using a targeted high performance liquid chromatography-tandem mass spectrometry (HPLC-MS/MS) method as described by Gart et al. (2021) (71), though small adaptations were made regarding the homogenization (performed in 2-propanol instead of H_2_O). Briefly, hypothalamic samples were thus first homogenized in 2-propanol as described in Method section 2.8. 200uL of this homogenate was then mixed with 1mL MeOH containing a mixture of internal standards (d4-LTB_4_, d8-15-HETE, d4-PGE_2_, d5-DHA, all Caymann Chemical) and 200uL water. Samples were incubated for 20 minutes at -20°C and spun down (16.100 x g, 4°C). Supernatant was transferred to a 15mL tube and diluted with 7.5mL water and acidified to pH 3.5 with formic acid. Lipids were then extracted using solid-phase extraction (SPE), the eluate was dried at 40°C under a stream of nitrogen and reconstituted in 200uL 40% MeOH. Oxylipin content of the samples was then analyzed using a Shimadzu LC-system coupled to a Sciex Qtrap 6500. For a detailed description of the method please see (71).

### 2.10 Downstream analysis lipid and oxylipin dataset

Data analysis was performed using Excel, Metaboanalyst (5.0) (72) and R4.0.3. For the broad lipidomics and oxylipin datasets, area normalization was applied (lipid species/sum of all lipids in the sample) and all lipid species with more than 20 missing values were excluded, except when this was specific for one of the experimental groups. For the broad lipidomic dataset specifically, the coefficient of variation (CoV, standard deviation/mean*100) was calculated for the quality controls for each lipid species. Species with CoV larger than 10% were excluded from the analysis since this is an indication for inaccurate measurement. For both datasets generalized logarithmic transformation (gLog) and pareto scaling was applied and remaining missing values were substituted by 1/5 of the lowest value measured for that species/derivative using MetaboAnalyst (5.0). Principal component analysis was carried oud to identify possible outliers and major effects in the data. Three outliers were detected in the broad lipidomic dataset and excluded from analysis. See **Table 1** for the final sample size per experimental group.

Normality was checked using Kolmogorov-Smirnov test (R package “domtools”). Three-way ANOVA was used to analyze lipid classes. In case of significant interaction effects, *post hoc* analyses were performed using Tukey’s *post hoc* test. Paired students T-test or the nonparametric alternative Mann Whitney U test (R4.0.3) was used for pairwise comparisons within lipid species and oxylipins. A p-value < 0.05 was considered significant. The R packages “ggplot”, “ComplexHeatmap”, “VennDiagram” and “Corrplot” were used to create boxplots, heatmaps, venn diagrams and correlation plots respectively. Because of the exploratory nature of this study we did not correct for multiple testing.

## 3 Results

In our experimental design we have three predictor variables: condition (*CTL/ELS*), diet (*HRD/LRD*) and second-hit (*SAL/LPS*), leading to eight experimental groups: *CTL-HRD-SAL, ELS-HRD-SAL, CTL-LRD-SAL, ELS-LRD-SAL, CTL-HRD-LPS, ELS-HRD-LPS, CTL-LRD-LPS, ELS-LRD-LPS* (**Table 1**). Considering the complexity of this design (**Figure 1A**), and in order to describe and disentangle the effects of ELS and of those of the protective effects of the diet on brain lipid profile, we studied first the baseline long-term effects of the early dietary PUFA ratio, second how ELS impacts the brain lipid profile depending on the diet and finally how the early-life environments (ELS and diet) impact LPS induced alterations in the lipid profile.

### 3.1 Effects of ELS, dietary ω6/ω3 ratio and LPS on bodyweight and plasma corticosterone

To assess how ELS and/or the early diet affect peripheral physiology, we assessed bodyweight (at P9 and in adulthood) and plasma corticosterone under basal conditions and in response to LPS. *ELS* reduced bodyweight gain in pups between P2-P9 (**Supplementary Figure 1A**; two-way ANOVA: condition F1,22 = 10.173, p=0.004, diet F1,22 = 0.103, p=0.751, interaction F1,22 = 0.207, p=0.654) confirming previous findings from our group (28, 66). ELS and diet did not affect food intake in dams from P2-P9 (**Supplementary Figure 1B**; two-way ANOVA: condition F1,22 = 4.241, p=0.051, diet F1,22 = 0.076, p=0.786, interaction F1,22 = 0.311, p=0.583). To assess the effect of *LPS* on bodyweight and plasma CORT levels, these parameters were measured before and 24 hours following *LPS* injections. No baseline differences in bodyweight were observed between groups before *LPS* treatment (**Supplementary Figure 1C**; two-way ANOVA: condition F1,20.525 = 3.483, p=0.076, diet F1,22.393 = 2.654, p=0.117, interaction F1,21.701 = 0.037, p=0.849). 24 hours after *LPS* treatment, a main effect of condition, treatment and an interaction effect between condition and treatment were detected for bodyweight. *LPS* and *ELS* decreased bodyweight, but the effect of *LPS* on bodyweight depended on condition and diet (**Supplementary Figure 1D**; three-way ANOVA: condition F1,39.873 = 8.049, p=0.007, diet F1,35.866 = 3.026, p=0.091, treatment F1,67.586 = 13.191, p=0.001, interaction condition*treatment F1,68.358 = 6.880, p=0.011, interaction condition*diet*treatment F1,75.321 = 4.449, p=0.038, no other interaction effects). *LPS* increased plasma CORT levels 24 hours after treatment without further modulation by condition or diet (**Supplementary Figure 1E**; three-way ANOVA: condition F1,75 = 0.042, p=0.839, diet F1,75 = 0.540, p=0.465, treatment F75 = 414.511, p<0.001, no interaction effects).

In summary, ELS, independent of diet, reduced bodyweight gain between P2 and P9 while no bodyweight differences were detected in adulthood under basal conditions. LPS reduced bodyweight which was dependent on both ELS and early diet. LPS increased plasma CORT levels independent of ELS and early diet.

### 3.2 Exposure to ELS exacerbates LPS-induced levels of IL-6, CXCL1 and CCL2

In order to understand if and how our early-life manipulations (ELS and diet) modulate the peripheral inflammatory response upon *LPS* injection, we next assessed plasma cytokine levels. As expected, *LPS* increased plasma levels of all measured cytokines, chemokines and growth factors (main effect of treatment for TNFα, IFN-γ, IL-1β, IL-10, IL-4, IL-6, CXCL1, CCL2, M-CSF, GM-CSF, VEGF) (**Figure 1B**), with the largest increase observed for IL-6 and CXCL1, and the smallest for IL-4 and GM-CSF (**Supplementary Table S1**). A main effect of condition, and an interaction effect between condition and treatment were observed for IL-6, CXCL1, and CCL2, with both ELS and LPS increasing these cytokines/chemokines. Further *post hoc* testing revealed that CXCL1 plasma levels in *ELS-HRD-LPS* were significantly higher as compared to *CTL-HRD-LPS* (p=0.008). No main effects of diet on cytokine expression levels were observed. However, for IL-4, an interaction between condition, diet and treatment was observed (**Supplementary Table S1**).

In summary, *LPS* induced a strong plasma cytokine response, which was exacerbated by *ELS* for the cytokine IL-6 and the chemokines CCL2 and CXCL1. Early diet (*HRD/LRD*) modulated IL-4 levels in interaction with *ELS* and *LPS*, with specifically the LRD decreasing the *LPS* induced increase in IL-4 in *CTL* mice, but not in *ELS* exposed mice.

### 3.3 Long-lasting effects of ELS and early dietary ω6/ω3 PUFA ratio on hypothalamic lipid profile at basal state and in response to LPS

**Figure 2:**
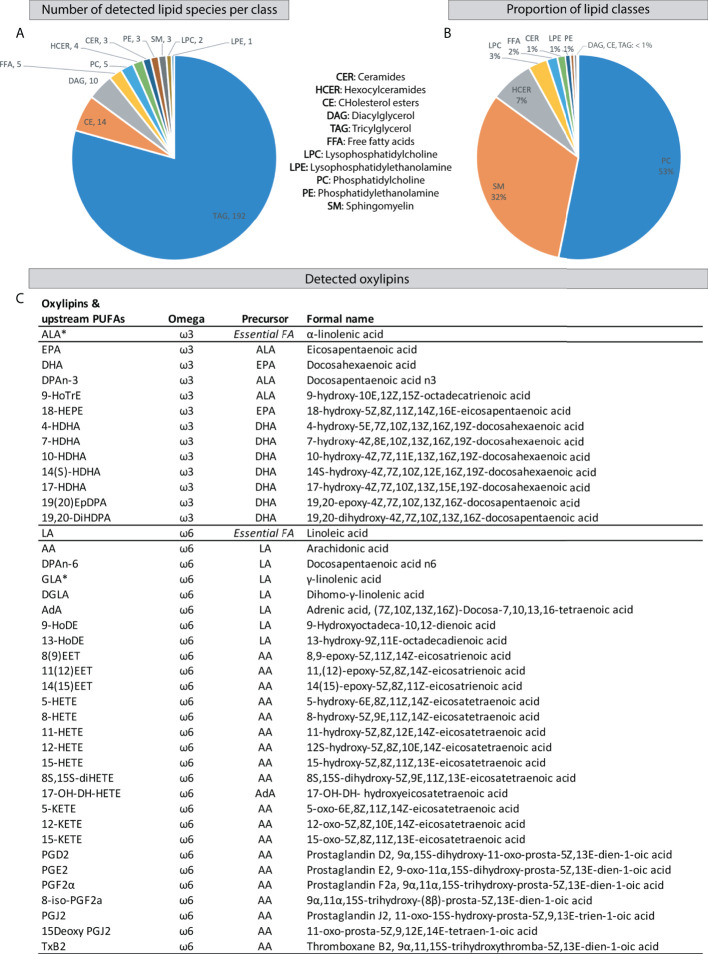
Detected hypothalamic lipids and oxylipins by HPLC-MS/MS. **(A)** Number of detected lipid species per class. **(B)** Proportion of hypothalamic lipid classes. **(C)** Detected oxylipins and their upstream PUFAs. HPLC-MS/MS, High Performance Liquid Chromatography-tandem Mass Spectrometry; FA, fatty acid; PUFAs, polyunsaturated fatty acids; *: Our methodology could not distinguish between the essential ω3 α-linolenic acid and ω6 γ-linolenic acid due to co-elution and identical molecular weight.

In order to assess if and how ELS impacts the brain lipidomic and oxylipin profile and whether the early-diet modulates such ELS-induced effects, we analysed the hypothalamic lipids and oxylipin profile in adulthood under basal conditions and in response to LPS. The curated lipidomics dataset contained included 242 lipid species from 11 lipid classes including ceramides (CER), hexosylceramides (HCER), cholesterol esters (CE), diacylglycerols (DAG), free fatty acids (FFA), lysophosphatidylcholine (LPC), lysophosphatidylethanolamine (LPE), phosphatidylcholine (PC), phosphatidylethanolamine (PE), sphingomyelin (SM), and triacylglycerol (TAG) (**Figure 2A**, **Supplementary Table S2**). PC’s and SM’s were most abundantly present in our analyses (**Figure 2B**). The curated oxylipin dataset contained 41 compounds (**Figure 2C**). Of note, our methodology could not distinguish between the essential ω3 PUFA α-linolenic acid (ALA) and the ω6 PUFA γ-linolenic acid (GLA) due to co-elution and identical molecular weight.

First we will describe the effects of *ELS*, *diet* and *LPS* on lipid classes followed by the effects of these parameters on distinct lipid species and oxylipins.

#### 3.3.1 Lipid classes

**Figure 3:**
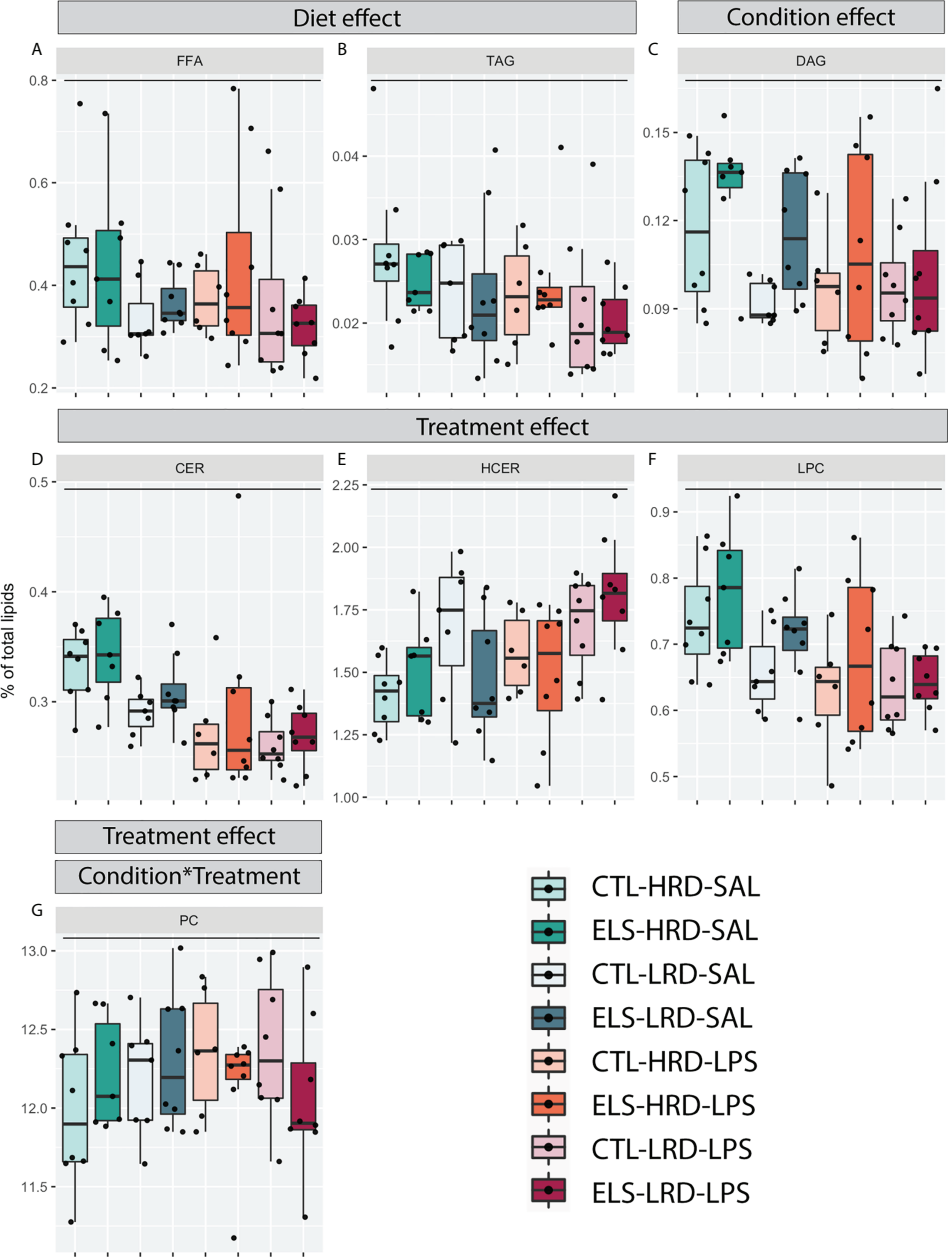
Effects of ELS, early dietary ω6/ω3 ratio on hypothalamic lipid classes. **(A-G)** Hypothalamic lipid classes with significant changes as tested by three-way ANOVA. **(A, B)** Main effect of diet for FFA and TAG. **(C)** Main effect of condition for DAG. **(D-G)** Main effect of treatment for CER, HCER, LPC and PC. **(G)** Interaction effect (Condition*Treatment) between condition and treatment for PC. CTL, control; ELS, early-life stress; HRD, high ω6/ω3 ratio diet; LRD, low ω6/ω3 ratio diet; LPS, lipopolysaccharide; ANOVA, analysis of variance; TAG, triacylglycerols; FFA, free fatty acids; DAG, diacylglycerols; CER, ceramides; HCER, hexosylceramide; LPC, lysophosphatidylcholine; PC, phosphatidylcholine. *: significant interaction effect, p < 0.05.

A main effect of diet was detected for FFA and TAG, with a reduction for both classes in mice fed the LRD (**Figures 3A, B**; three-way ANOVA: FFA: condition F1,52 = 0.013, p=0.911, diet F1,52 = 4.441, p=0.040, treatment F1,52 = 0.48, p=0.491; TAG: condition F1,52 = 0.040, p=0.842, diet F1,52 = 5.493, p=0.023, treatment F1,52 = 0.68, p=0.415). For DAG a main effect of condition was detected (**Figure 3C**; three-way ANOVA condition F1,52 = 4.642, p=0.036, diet F1,52 = 0.739, p=0.394, treatment F1,52 = 3.660, p=0.061). A main effect of treatment was detected for CER, HCER and LPC (**Figure 3D-F**; three-way ANOVA: CER: condition F1,52 = 0.595, p=0.444, diet F1,52 = 3.6, p=0.063, treatment F1,52 = 17.2, p=0.000; HCER: condition F1,52 = 0.402, p=0.444, diet F1,52 = 3.403, p=0.081, treatment F1,52 = 5.77, p=0.020; LPC: condition F1,52 = 2.21, p=0.143, diet F1,52 = 3.537, p=0.066, treatment F1,52 = 12.94, p=0.001). For PC, next to a main effect of treatment, an interaction effect between condition and treatment was detected (**Figure 3G**; three-way ANOVA: condition F1,52 = 0.083, p=0.775, diet F1,52 = 0.23, p=0.632, treatment F1,52 = 13.11, p=0.001, condition*treatment F1,52 = 4.05, p=0.049).

In summary, the *LRD* reduced levels of FFA and TAG both under basal conditions and in response to *LPS*. *ELS* increased levels of DAG. *LPS* increased levels of HCER while it decreased CER and LPC. For PC, the effect of *LPS* was dependent on previous exposure to *ELS*.

#### 3.3.2 Lipid species and oxylipins under basal conditions

**Figure 4:**
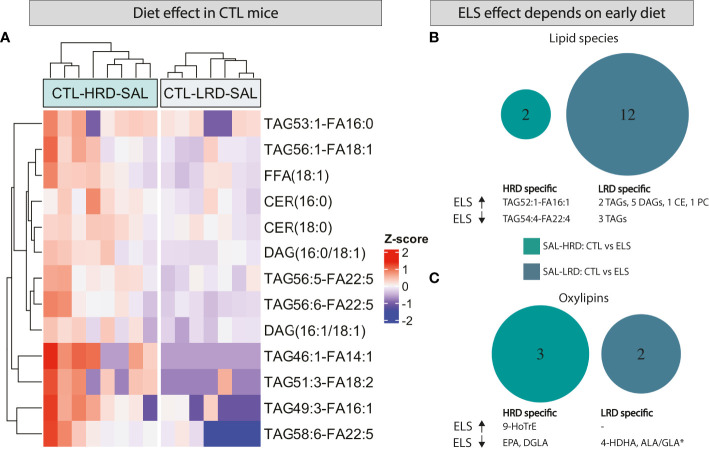
Both ELS and early dietary ω6/ω3 ratio modulate later-life hypothalamic lipid species and oxylipins, in interaction with each other. **(A)** Heatmap depicting modulation of hypothalamic lipid species by early dietary ω6/ω3 ratio **(B, C)** Venn diagram depicting diet dependent effects of ELS on lipid species **(B)** and oxylipins **(C)**. CTL, control; ELS, early-life stress; SAL, saline, LPS, lipopolysaccharide; HRD, high ω6/ω3 ratio diet; LRD, low ω6/ω3 ratio diet.

All significant changes for the considered contrasts are described in **Table 3** for lipid species and **Table 4** for oxylipins. In addition, the raw lipid values per experimental group are provided in **Supplementary Table S3** for lipid species and **Supplementary Table S4** for oxylipins. To determine the effect of diet on lipid profiles under basal conditions, *CTL* saline injected mice fed either the *HRD* or *LRD* were compared (*CTL-SAL: HRD vs LRD*). Thirteen significantly altered lipid species were detected ^(2 CERs,2 DAGs,1 FFA and 7 TAGs)^ which were all reduced in mice fed the *LRD* versus those fed the *HRD* (**Figure 4A**). There was no diet effect on oxylipins in *CTL* mice under basal conditions. Next, we tested the effect of condition on lipid species and oxylipins and whether this was dependent on the diet by comparing *ELS* with *CTL* mice that were fed either the *HRD* or *LRD* (*SAL-HRD*: *CTL* vs *ELS* and *SAL-LRD: CTL* vs *ELS*; **Figure 4B**). Under both dietary conditions, *ELS* impacts lipid species and oxylipins and these *ELS*-induced lipid profiles are unique depending on the diet. For lipid species under the *HRD*, *ELS* altered 2 lipid species which were both TAGs ^(increased: TAG54:4-FA22:4; decreased TAG52:1-FA16:1)^, while under the *LRD ELS* altered a total of 12 lipid species ^(increased: 2 TAGs,5 DAGs,1 CE,one PC; decreased: 3 TAGs)^ (**Figure 4B**). For oxylipins, under the *HRD*, *ELS* increased the ω3 derivative 9-HoTrE and decreased the ω6 fatty acid DGLA and the ω3 PUFA EPA. Under the *LRD*, *ELS* decreased the ω3 derivative 4-HDHA and ALA/GLA (**Figure 4C**).

**Table 3:** Significant effects of early dietary ω6/ω3 ratio, ELS and LPS on lipid species.

**A) Diet effect on lipid species in CTL mice under basal conditions**			
**Lipid species**	**p-value**	**Fold change**	**Direction**
Diet effect in CTL mice (CTL-SAL: HRD versus LRD)			
CER(16:0)	0,030	0,299	high > low
CER(18:0)	0,011	0,225	high > low
DAG(16:0/18:1)	0,017	0,290	high > low
DAG(16:1/18:1)	0,046	0,321	high > low
FFA(18:1)	0,037	0,352	high > low
TAG46:1-FA14:1	0,007	1,113	high > low
TAG49:1-FA17:0	0,036	0,790	high > low
TAG49:3-FA16:1	0,029	0,936	high > low
TAG51:3-FA18:2	0,038	0,868	high > low
TAG53:1-FA16:0	0,042	0,396	high > low
**B) ELS effect on lipid species in mice fed the HRD or LRD, under basal conditions**			
ELS effect in mice fed the HRD (HRD-SAL: CTL versus ELS)			
TAG52:1-FA16:1	0,035	0,947	CTL > ELS
TAG54:4-FA22:4	0,029	-0,336	ELS > CTL
ELS effect in mice fed the LRD (LRD-SAL: CTL versus ELS)			
TAG51:0-FA16:0	0,048	-0,257	ELS > CTL
TAG52:5-FA20:5	0,043	-0,724	ELS > CTL
TAG56:4-FA16:0	0,048	0,172	CTL > ELS
TAG56:4-FA18:1	0,022	0,301	CTL > ELS
TAG56:5-FA16:0	0,041	0,257	CTL > ELS
DAG(16:0/18:1)	0,024	-0,225	ELS > CTL
DAG(16:1/18:1)	0,049	-0,280	ELS > CTL
DAG(16:1/20:4)	0,011	-0,724	ELS > CTL
DAG(18:0/18:2)	0,001	-0,490	ELS > CTL
DAG(18:1/18:1)	0,043	-0,327	ELS > CTL
CE(22:5)	0,021	-0,370	ELS > CTL
PC(16:0/18:2)	0,043	-0,517	ELS > CTL
**C) LPS effect on lipid species in CTL mice fed the HRD or LRD**			
LPS effect in CTL mice fed the HRD (CTL-HRD: SAL versus LPS)			
CER(16:0)*	0,031	0,296	sal > lps
CER(18:0)	0,024	0,355	sal > lps
CE(20:4)*	0,043	-0,404	lps > sal
DAG(16:0/18:2)	0,035	-0,271	lps > sal
DAG(16:0/20:5)	0,042	-0,777	lps > sal
HCER(20:0)	0,037	-0,303	lps > sal
HCER(22:0)	0,041	-0,268	lps > sal
LPC(18:1)	0,002	0,493	sal > lps
PC(16:0/18:2)	0,039	-0,242	lps > sal
TAG52:3-FA20:3	0,020	-0,279	lps > sal
TAG54:4-FA20:3	0,001	-0,365	lps > sal
TAG54:5-FA20:3	0,002	-1,145	lps > sal
LPS effect in CTL mice fed the LRD (CTL-LRD: SAL versus LPS)			
CER(18:0)	0,023	0,183	sal > lps
CE(18:0)*	0,002	0,608	sal > lps
DAG(16:0/18:2)	0,019	-0,836	lps > sal
DAG(16:0/20:5)	0,017	-0,948	lps > sal
DAG(16:1/20:4)*	0,011	-0,773	lps > sal
**C) LPS effect on lipid species in CTL mice fed the HRD or LRD**			
LPS effect in CTL mice fed the LRD (CTL-LRD: SAL versus LPS)			
DAG(18:1/18:2)*	0,004	-0,517	lps > sal
PC(16:0/18:2)	0,001	-0,893	lps > sal
PC(18:0/18:2)*	0,004	-1,012	lps > sal
TAG42:0-FA16:0*	0,048	0,574	sal > lps
TAG48:0-FA14:0*	0,040	0,324	sal > lps
TAG48:0-FA16:0*	0,027	0,306	sal > lps
TAG48:1-FA14:0*	0,040	0,467	sal > lps
TAG48:1-FA16:0*	0,025	0,529	sal > lps
TAG48:1-FA18:1*	0,017	0,438	sal > lps
TAG48:2-FA16:0*	0,038	0,605	sal > lps
TAG50:0-FA16:0	0,014	0,324	sal > lps
TAG50:0-FA18:0*	0,008	0,265	sal > lps
TAG50:1-FA16:0*	0,028	0,409	sal > lps
TAG50:1-FA18:1*	0,035	0,349	sal > lps
TAG50:2-FA18:0*	0,044	0,862	sal > lps
TAG50:2-FA18:1*	0,037	0,515	sal > lps
TAG52:0-FA16:0*	0,003	0,307	sal > lps
TAG52:0-FA18:0	0,000	0,440	sal > lps
TAG52:1-FA18:0*	0,011	0,364	sal > lps
TAG52:1-FA18:1*	0,015	0,370	sal > lps
TAG54:4-FA20:3	0,040	-0,272	lps > sal
TAG54:5-FA22:4*	0,005	0,544	sal > lps
TAG56:3-FA18:0*	0,009	0,253	sal > lps
TAG56:4-FA18:0	0,016	0,328	sal > lps
TAG56:5-FA16:0*	0,046	0,227	sal > lps

*Unique LPS induced effect per contrast.

**Table 4:** Significant effects of dietary ω6/ω3 ratio, ELS and LPS on oxylipins.

A) ELS effect on oxylipins in mice fed the HRD or LRD, under basal conditions				
Oxylipin	Pathway	p-value	Fold change	Direction
ELS effect in mice fed the HRD (HRD-SAL: CTL versus ELS)				
EPA	ω3	0,003	0,410	CTL > ELS
9-HoTrE	ω3	0,029	-0,735	ELS > CTL
DGLA	ω6	0,027	0,363	CTL > ELS
ELS effect in mice fed the LRD (LRD-SAL: CTL versus ELS)				
4-HDHA	ω3	0,025	0,420	CTL > ELS
ALA/GLA*	ω3 or ω6*	0,032	0,299	CTL > ELS
**B) LPS effect on oxylipins in CTL mice fed the HRD or LRD**				
LPS effect in CTL mice fed the HRD (CTL-HRD: SAL versus LPS)				
PGE2	ω6	0,000	-0,756	lps > sal
LPS effect in CTL mice fed the LRD (CTL-LRD: SAL versus LPS)				
DPAn-3*	ω3	0,024	0,612	sal > lps
10-HDHA*	ω3	0,030	0,421	sal > lps
AA*	ω6	0,022	0,569	sal > lps
5-HETE*	ω6	0,005	0,559	sal > lps
8-HETE*	ω6	0,029	0,546	sal > lps
12-HETE*	ω6	0,015	0,760	sal > lps
5-KETE*	ω6	0,017	0,500	sal > lps
12-KETE*	ω6	0,045	0,675	sal > lps
15-KETE*	ω6	0,010	0,578	sal > lps
TxB2*	ω6	0,020	0,587	sal > lps
PGF2a*	ω6	0,028	0,550	sal > lps
PGE2	ω6	0,002	-0,840	lps > sal
**C) LPS effect on oxylipins in ELS mice fed the HRD or LRD**				
LPS effect in CTL mice fed the HRD (CTL-HRD: SAL versus LPS)				
PGE2	ω6	0,000	-0,756	lps > sal
LPS effect in CTL mice fed the LRD (CTL-LRD: SAL versus LPS)				
DPAn-3*	ω3	0,024	0,612	sal > lps
10-HDHA*	ω3	0,030	0,421	sal > lps
AA*	ω6	0,022	0,569	sal > lps
5-HETE*	ω6	0,005	0,559	sal > lps
8-HETE*	ω6	0,029	0,546	sal > lps
12-HETE*	ω6	0,015	0,760	sal > lps
5-KETE*	ω6	0,017	0,500	sal > lps
12-KETE*	ω6	0,045	0,675	sal > lps
15-KETE*	ω6	0,010	0,578	sal > lps
TxB2*	ω6	0,020	0,587	sal > lps
PGF2a*	ω6	0,028	0,550	sal > lps
PGE2	ω6	0,002	-0,840	lps > sal

*Our methodology could not distinguish between ALA and GLA due to co-elution and identical molecular weight.

*Unique LPS induced effects per contrast.

*Unique LPS induced effect per experimental group.

In summary, under basal conditions, the *LRD* reduced several lipid species such as FFA, TAG, DAG and CER while it did not affect the presence of oxylipins directly. Exposure to *ELS* impacts an entirely different set of lipid species and oxylipins depending on the early diet, with the *LRD* leading to a larger impact of ELS on lipid species with altered TAGs and increased DAGs, CE and PC.

#### 3.3.3 Lipid species and oxylipins in response to LPS

**Figure 5:**
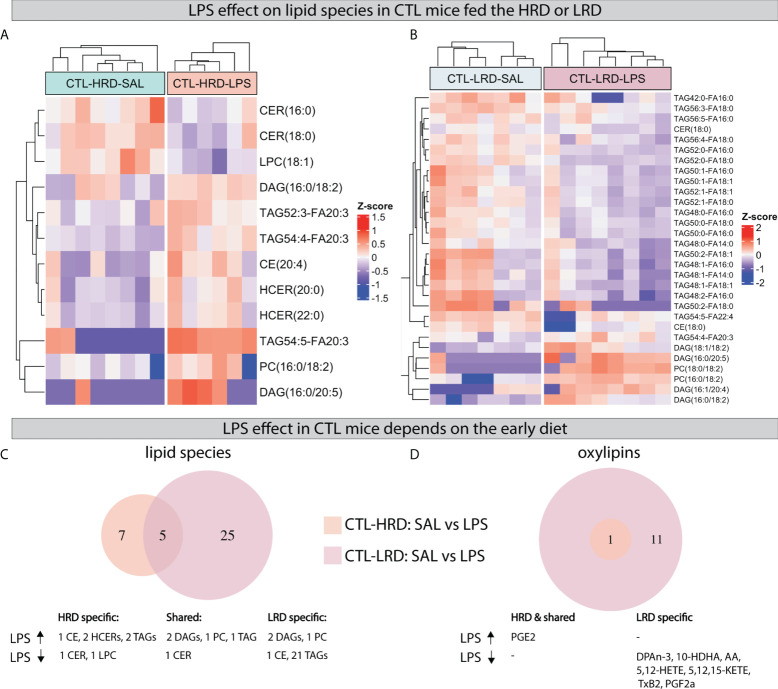
LPS induced changes in hypothalamic lipid species and oxylipins depend on early dietary ω6/ω3 ratio. **(A, B)** Heatmaps depicting LPS induced changes in lipid species in CTL mice fed the HRD **(A)** or LRD **(B)**. **(C, D)** Venn diagrams depicting the diet dependent effects of LPS in CTL mice on lipid species **(C)** and oxylipins **(D)**. CTL, control; ELS, early-life stress; SAL, saline; LPS, lipopolysaccharide; HRD, high ω6/ω3 ratio diet; LRD, low ω6/ω3 ratio diet.

Next we tested the effect of *LPS* on lipid species and oxylipins in *CTL* mice and whether this was dependent on diet by comparing saline injected *CTL* mice to *LPS* injected *CTL* mice fed either the *HRD* or *LRD* (*CTL-HRD: SAL* vs *LPS* and *CTL-LRD: SAL* vs *LPS*). *LPS* resulted in several changes in the measured hypothalamic lipid species 24 hours later (**Figure 5A, B**). The majority of *LPS* induced changes in lipid species were unique for each diet and more species were affected in mice fed the *LRD*. Under the *HRD*, *LPS* altered seven species ^(increased: 1 CE,2 HCERs,2 TAGs; decreased: 1 CER,1 LPC)^. Under the *LRD*, *LPS* altered 25 species ^(increased: 2 DAGs,1 PC; LPS decreased: 1 CE and 21 TAGs)^. Only five *LPS* responsive lipid species were shared between *CTL* mice fed the *HRD* or *LR* ^(increased: 2 DAGs,1 PC and 1 TAG; decreased: 1 CER)^ (**Figure 5C**).

Regarding oxylipins, an increase was observed for prostaglandin E_2_ (PGE_2_) in all experimental groups regardless of early life condition or diet. This was the only significant *LPS-*induced change in CTL mice fed *HRD*, while, in *CTL* mice fed the *LRD*, an additional 11 *LPS* responsive oxylipins were detected which were all reduced by *LPS* (DPAn-3, 10-HDHA, AA, 5-HETE, 8-HETE, 12-HETE, 5-KETE, 12-KETE, 15-KETE, TxB2, PGF2α; **Figure 5D**).

**Figure 6:**
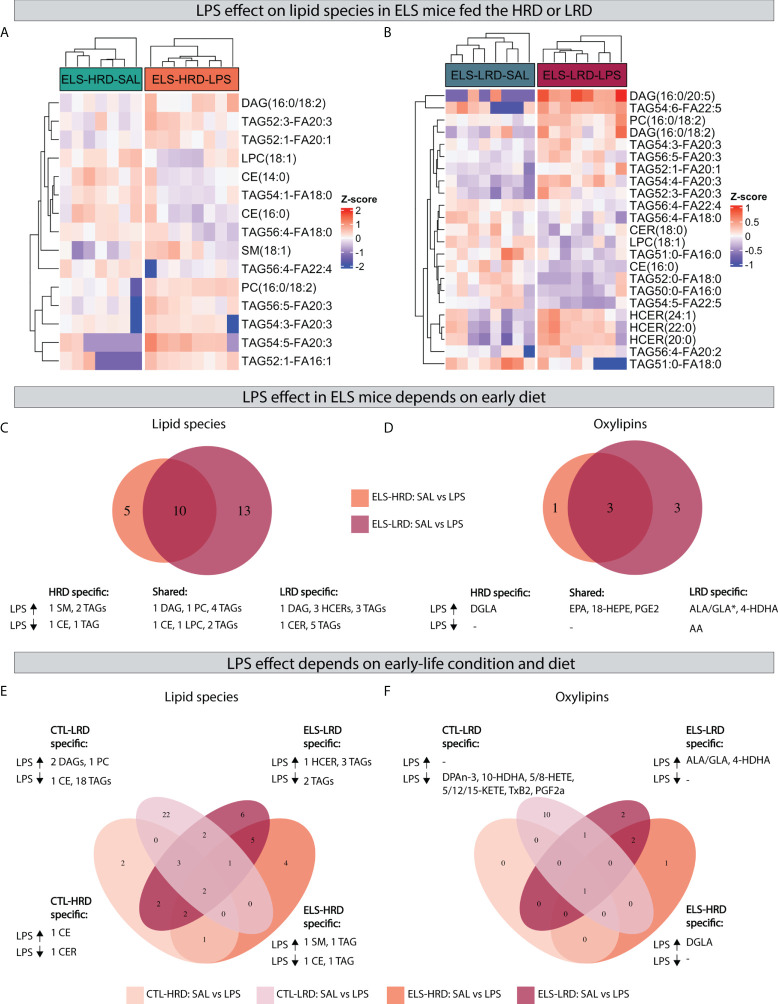
Effects of an acute LPS challenge on hypothalamic lipid species depend on previous exposure to ELS and dietary ω6/ω3 ratio. AB) Heatmaps depicting LPS effects in mice exposed to ELS fed either the HRD **(A)** or LRD **(B)**. **(C, D)** Venn diagram depicting diet dependent effects of LPS in ELS exposed mice on lipid species **(C)** and oxylipins **(D)**. **(E, F)** quad venn diagram depicting condition and diet dependent effects of LPS on lipid species **(E)** and oxylipins **(F)**. CTL, control; ELS, early-life stress; SAL, saline; LPS, lipopolysaccharide; HRD, high ω6/ω3 ratio diet; LRD, low ω6/ω3 ratio diet.

When analyzing the impact of *LPS* in *ELS* exposed mice, comparing (*ELS-HRD: SAL* vs *LPS and ELS-LRD: SAL* vs *LPS*) we found that *LPS* leads to a specific set of changes in lipid species and oxylipins in mice fed either the *HRD* (**Figure 6A**) or the *LRD* (**Figure 6B**) diet. Considering the uniquely altered lipid species, under the *HRD*, *LPS* altered five lipid species in *ELS* exposed mice ^(increased: 1 SM and 2 TAGs; decreased: 1 CE and 1 TAG)^, while under the *LRD*, *LPS* altered 13 lipid species ^(increased: 1 DAG,3 HCERs and 3 TAGs; decreased: 1 CER,and 5 TAGs)^. In addition 10 lipid species were found to be altered under both dietary conditions ^(increased: 1 DAG,1 PC,4 TAGs; decreased: 1 CE,1 LPC and 2 TAGs)^ (**Figure 6C**).

Concerning oxylipins, *LPS* led to several changes in *ELS* exposed mice, which was dependent on the early diet. Considering the uniquely altered oxylipins: under the *HRD* LPS increased DGLA and under the *LRD* LPS increased 4-HDHA and ALA/GLA while it decreased AA. In addition, there were three shared oxylipins increased by *LPS* in *ELS* mice independent of diet (EPA, 18-HEPE and PGE_2_) (**Figure 6D**).

In order to investigate whether the *LPS* response in *ELS* mice is different from *CTL* mice, and to test whether this was dependent on the diet, we compared *LPS* responsive lipid species and oxylipins in *CTL* mice to those of *ELS* mice, in mice fed either the *HRD* or *LRD* (**Figures 6E, F**). Exclusively in *CTL* mice fed the *HRD*, *LPS* altered two lipid species ^(1 CER and 1 CE)^. In *CTL* mice fed the *LRD* the highest number of exclusively regulated lipid species was detected ^(1 CE,2 DAGs,1 PC and 18 TAGs)^. In *ELS* mice fed the *HRD*, four exclusive lipid species were detected ^(1 CE,1 SM,2 TAGs)^. In *ELS* mice fed the *LRD*, six lipid species were regulated exclusively ^(1 HCER,5 TAGs)^ (**Figure 6E**).

Concerning oxylipins in response to *LPS*, no *CTL-HRD* specific changes were detected, while in *CTL* mice fed the *LRD*, the levels of 10 oxylipins were reduced by LPS in this experimental group only (DPAn-3, 10-HDHA, 5-HETE, 8-HETE, 5-KETE, 12-KETE, 15-KETE, TxB2, PGF_2α_). In *ELS* mice fed the *HRD* specifically DGLA was upregulated and in ELS mice fed the *LRD* ALA/GLA and 4-HDHA (**Figure 6F**).

In summary, 24 hours after an *LPS* injection, several changes in hypothalamic lipid species and oxylipins were observed which were mostly unique dependent on both ELS and diet. Generally, in mice fed the *LRD* more *LPS* induced changes were detected, both in lipid species (decrease in TAGs) and oxylipins (decrease of ω6 derived oxylipins).

### 3.5 Correlations

**Figure 7:**
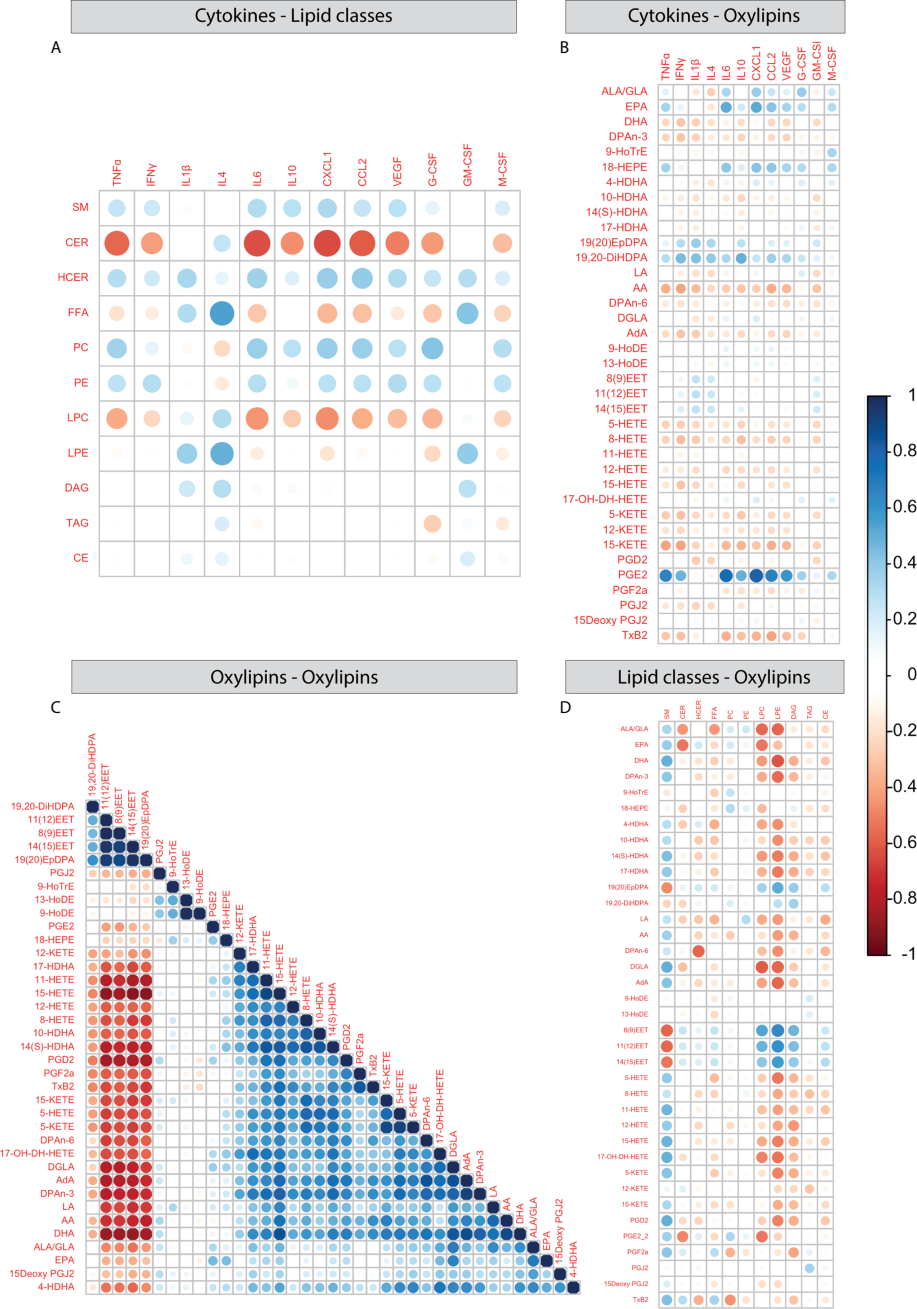
Correlations between plasma cytokines and hypothalamic lipid classes and oxylipins. **(A–D)** Correlation plots depicting Pearson correlation between plasma cytokines and hypothalamic lipid classes **(A)**, plasma cytokines and hypothalamic oxylipins **(B)**, oxylipins and oxylipins **(C)**, oxylipins and lipid classes **(D)**.

In order to better understand the relationship between peripheral plasma cytokines and hypothalamic lipid profiles, we correlated cytokine levels with hypothalamic lipid classes (**Figure 7A**) and oxylipins (**Figure 7B**). Negative correlations were detected between several cytokines (TNFα, INFγ, IL-6, IL-10, CXCL1, CCL2, CEGF, G-CSF) and CER and IL-4 correlated positively with FFA and LPE. For oxylipins, strong positive correlations were detected between PGE_2_ and multiple cytokines (TNFα, INFγ, IL-6, IL-10, CXCL1, CCL2, VEGF).

Next, we correlated oxylipins to each other using expression levels over all experimental groups. One large and one smaller cluster appeared. The largest cluster 1 consists of mainly AA derivatives together with several ω3 and ω6 PUFAs. Cluster 2 consisted of the ω3 derivatives epoxy- and dihydroxy docosapentaenoic acids (DPA) (19,20-diHDPA, 19(20)EpDPA) and three epoxyyeicosatrienoic acids (11(12)EET, 8(9)EET and 13(15)EET). In addition, 9- and 13-HoDE correlated with each other and there were a few oxylipins that did not correlate with any other oxylipin (PGJ_2_, PGE_2_, 9-HoTrE) (**Figure 7C**). When testing oxylipin-oxylipin correlations per experimental group, most patterns were similar to what was observed when collapsing all groups (**Supplementary Figure S2**), however *ELS* mice fed the *HRD* and injected with *SAL* showed the most distinct profile (**Supplementary Figure S2B**).

Lastly, when investigating correlations between expression of lipid classes and oxylipins several correlations were detected between LPC, LPE and multiple oxylipins, CER correlated with ALA/GLA and EPA and SM conversely correlated with EETs (**Figure 7D**).

Together these findings indicate that peripheral inflammation relates to hypothalamic lipid profiles in a few cases, with the strongest example being the positive correlation between plasma cytokines and hypothalamic PGE_2_ levels. When looking at how oxylipins relate to each other, we observed that the majority of measured oxylipins and upstream PUFAs correlate with each other. The oxylipin-oxylipin correlation profile was most distinct for ELS mice fed the HRD injected with saline as compared to the other experimental groups, indicating altered oxylipin metabolism in these mice.

## 4 Discussion

In this study, we aimed to gain further insights into whether modulation of the brain lipid profile may be one of the mechanisms contributing to the i) ELS-induced increased risk for psychopathology and cognitive decline and ii) protective effects of dietary PUFAs against the ELS-induced deficits. Here we provide evidence for long-term effects of ELS and an early dietary intervention, based on altering the ratio of dietary ω6/ω3 PUFAs, on plasma cytokines as well as hypothalamic lipid and oxylipin profiles, both under basal conditions as well as in response to an inflammatory challenge in adulthood. Remarkably, depending on the early diet, ELS led to entirely distinct lipid and oxylipin changes and similarly the LPS-induced alterations also depended on ELS and diet. Our findings suggest that brain lipid imbalance in adulthood can have an early-life origin and that it potentially contributes to the later life ELS-induced increased risk for psychopathologies and cognitive decline as well as to the long-term protective effects of dietary PUFAs.

### 4.1 ELS exacerbates the LPS induced increase in plasma levels of IL-6, CXCL1 and CCL2

ELS increased levels of IL-6, CXCL1 and CCL2 and specifically exacerbated the LPS-induced response of these cytokines, suggesting priming of the immune response by ELS. These peripheral observations are in line with our earlier reported ELS-induced exaggerated neuro-immune response in the hippocampus (21, 28) suggesting an effect of ELS on both central as well as the peripheral immune system. In line with our results, ELS induced *via* daily maternal separation was shown to exacerbate the increase in plasma IL-6 following a single LPS challenge in adulthood (73) and similarly in humans, basal peripheral IL-6 levels were elevated in adults that were previously exposed to childhood trauma (74). We did not observe major effects of the diet on plasma cytokines in adulthood, only an interaction effect between ELS, diet and LPS on IL-4, suggesting a potential subtle modulation of the diet on the ELS and LPS induced effects on IL-4. Previously, a direct effect of dietary FA has been shown to protect against the increase in the LPS-induced pro-inflammatory plasma cytokines IL1ß, IL-6, and TNFα (75–77). A possible explanation for this discrepancy might lie in the relatively high dosage of LPS used in the present study and the specifics of our dietary intervention (which ended three months prior to cytokine analysis). This supports the idea that circulating levels of ω3 FA might need to be high at the time of LPS stimulus to exert a protective effect on plasma cytokine levels.

### 4.2 Early dietary ω6/ω3 ratio has long-term effect on hypothalamic lipid profile in adulthood

Early dietary ω6/ω3 ratio from P2 to P42 impacted the hypothalamic lipid profile on the long-term. The low ω6/ω3 ratio diet reduced levels of free fatty acids (FFA) and triacylglycerols (TAG) and several diacylglycerol (DAG) and ceramide (CER) species. To our knowledge we are the first to describe such long-term effects of early dietary PUFAs on brain lipid profile in adulthood. However, supporting the notion that dietary fatty acid can impact brain lipid levels, high-fat diet induces changes in the lipid profile of several brain regions in rodents (cortex, hippocampus, hypothalamus and olfactory bulb) and specifically increased DAG and TAG in the hypothalamus (78, 79). In line with the observed reduction of TAGs, in humans, high ω3 PUFA intake has been associated with lower levels of serum TAG (80, 81). The TAG-reducing effect of ω3 PUFAs might be related to their ability to downregulate or inhibit genes and enzymes involved in the synthesis of FA and TAG, and their affinity for peroxisome proliferator-activated receptor (PPAR) subtypes thereby stimulating lipid catabolism (82–84). Despite the fact that TAGs make up only a small fraction of the brain lipid pool in both rodents and humans (4), their role in the storage of lipid precursors and ability to release FFA suggests that small alterations can lead to functional outcomes. For example, elevated TAG, primarily as triacylglycerol-rich lipoproteins (TGRL), and the release of FFA by the lipolysis of TGRL can initiate inflammatory signaling pathways (85, 86), possibly contributing to pathologies with an inflammatory component (87, 88).

The low ω6/ω3 ratio diet also led to a reduction in two long-chain CER species (C16 and C18). Importantly, we were able to detect only C16, C18 and C24 ceramide species. Ceramides are the backbone of all complex sphingolipids that take part in fundamental cellular processes including cell proliferation, growth, differentiation, survival and apoptosis (89). They have also been implicated in neuroinflammatory signaling and increased levels have been associated with neurodegenerative disorders (90, 91). In addition, ceramides have been proposed to impact the fluidity of lipid rafts in synaptic membranes and contribute to reshaping synaptic structures, learning and memory (36). Indeed, reduced C16 ceramide levels in the hippocampus have been associated with learning and memory. More specifically, learning coincided with a decrease in the activity of sphingomyelinase (ASM), which catalyzes the turnover of sphingomyelin to ceramide. The stronger the decline in ASM activity, the better the learning (92). The low ω6/ω3 diet reduction in long-chain ceramide species might thus both lead to a more anti-inflammatory profile as well as contribute to changes in synaptic plasticity, thereby possibly contributing to its beneficial effects on learning and memory.

In summary, the early low ω6/ω3 diet reduces several hypothalamic lipids (TAG, FFA, DAG and long-chain CERs) seemingly leading to a more anti-inflammatory environment and possibly supporting synaptic plasticity. This data provides a possible mechanism *via* which the low dietary ω6/ω3 diet exerts its anti-inflammatory and pro-neuronal plasticity actions, also in the brain. Without directly affecting oxylipin levels, possibly due to the fact that our dietary intervention ended long time before (P42) the measurement of oxylipin levels and the absence of an inflammatory state, the early diet did impact the effects of ELS and LPS on oxylipins which will be discussed in the upcoming sections.

### 4.3 Exposure to ELS impacts lipid profile, dependent on early dietary ω6/ω3 ratio

ELS exposure increased the lipid class DAG in the adult hypothalamus independent of diet while at level of the lipid species the ELS-induced changes were dependent on the early dietary ω6/ω3 ratio. In mice fed the high ω6/ω3 diet previous ELS exposure altered only two TAG species while in mice fed the low ω6/ω3 diet ELS altered 12 lipid species including TAGs, DAGs, CE and PC.

Because we are the first to report on the effects of ELS on brain lipid profile, it is difficult to relate our findings to previous literature. Nonetheless, there is evidence that stress can impact on brain lipid composition. For example, similar to the long-term modulation that we observe, chronic unpredictable stress in adulthood has been shown to increase several lipids such as DAG, TAG and CER in the hippocampus and prefrontal cortex (93–95) as well as DAG precursors in the amygdala (96). DAGs serve as important second messengers, for example, DAG lipases (DAGLα and DAGLβ) convert DAG to the endocannabinoid 2-arachidonoylglycerol (2-AG) (97, 98), a bioactive compound playing crucial roles in synaptic signaling, axonal guidance and adult neurogenesis, processes known to be affected by ELS (13, 14). Indeed ELS has been shown to affect developmental endocannabinoid signaling in the hippocampus (99), but whether these effects are mediated by ELS-induced effects on DAG and DAG lipases remains to be investigated.

Specifically in mice fed the low ω6/ω3 diet, ELS also increased cholesterol ester CE(22:5) and phosphatidylcholine PC(16:0/18:2), both implicated in neuronal plasticity and neurodegenerative diseases. In fact, disruptions in cholesterol homeostasis and increased cholesterol esterification (synthesis of CE) have been reported for example in neurodegenerative diseases including Alzheimer’s disease (100). PC is a major component of the biological membrane accounting for approximately 45% of total phospholipids (101), thereby acting as an important component in membrane integrity, endocytosis and enzymatic activity and has been associated with learning and memory in rodents. The low ω6/ω3 diet was able to restore the ELS-induced cognitive decline and reduction in adult hippocampal neurogenesis (28). Thus, the increase in PC in ELS mice fed the low ω6/ω3 diet could possibly contribute to the beneficial effects of the diet on neuronal plasticity and learning and memory.

Similar to the effects of ELS on lipid classes and species, ELS-induced effects on PUFA’s and oxylipins were dependent on the early diet. Amongst others, under the high ω6/ω3 diet, ELS decreased the ω3 PUFA EPA, which was not the case in ELS exposed mice fed the low ω6/ω3 diet. Previously we have shown that the low ω6/ω3 diet from P2–P42 led to an increase in hippocampal levels of EPA both directly after the dietary intervention at P42 and into adulthood (28). This and the current data point towards long-lasting effects of an early low ω6/ω3 diet on brain ω3 EPA levels, which in the hypothalamus is specific for ELS exposed mice, possibly mediating the beneficial effects of the diet.

In summary, ELS leads to long-term changes in the brain lipid profile. Remarkably the changes in lipid profile are highly dependent on the early diet. In particular it appears that ELS mice fed the low ω6/ω3 diet differently from those fed the high ω6/ω3 present an overall anti-inflammatory and neuroplasticity promoting lipid profile. This might thus (at least in part) contribute to the mechanisms *via* which the low ω6/ω3 diet protects ELS-exposed mice against the long-lasting cognitive decline, as well as deficit in neurogenesis and increased neuroinflammation (21, 28).

### 4.4 LPS impacts the hypothalamic lipid profile, dependent on exposure to ELS and dietary ω6/ω3 ratio

To fully understand the long-term effects of early-life exposures (stress and diet), we studied these parameters not only under basal conditions but also in response to an LPS challenge in adulthood as a ‘second hit’ in order to unmask possible latent effects (21, 65). Inflammation induced *via* an acute LPS challenge led to several changes in hypothalamic lipid profiles 24 hours later. For example, LPS decreased the lipid classes CER and lysophosphotidylcholine (LPC) while it increased hexosylceramides (HCER) of which some were independent of early-life stress or diet, while others were modulated by them.

While lipids and their derivatives have been proven to be potent mediators of inflammation, there haven’t been many studies performed to reveal the effects of LPS on brain lipids. One previous study showed that LPS induced changes in lipid dynamics using desorption electrospray ionization mass spectrometry (DESI-MS) to image the distribution of lipids in the brain (102). Despite the limited amount of lipids detected with this technique, their data illustrates the involvement of brain lipids in LPS induced neuroinflammation. Using high-performance liquid chromatography mass spectrometry (HPLC-MS/MS), the effects of LPS on the brain lipidome has been studied in the context of an Alzheimer’s disease transgenic mouse model (APPswe/PS1dE9) (103). Low grade chronic LPS induced inflammation did not lead to changes in brain lipidome of wild type mice, while in the Alzheimer’s disease transgenic mice LPS induced several changes such as increases in DAG and alterations in LPC/LPE and PC/PE metabolism. The discrepancies between this and the current study most likely lies in the different LPS protocol, where Puris and colleagues (2021) applied chronic low dose LPS administration (500 µg/kg i.p. twice a week for four weeks, repeated twice) and a 5-week washout period between the last LPS injection and lipid analysis (103), we sacrificed the mice 24 hours after a single acute challenge of 5 mg/kg i.p. LPS injection.

In the current study LPS reduced LPC independent of ELS or diet. LPC is mainly derived from the turnover of PC and similarly a major component of the lipid bilayer with immunomodulatory functions, also in the brain (104). Several studies have revealed pro-inflammatory activities of LPC: activation of glial cells to produce proinflammatory cytokines, inducing demyelination *in vitro* and *in vivo* (105) and elevated LPC levels have been reported in several chronic diseases including multiple sclerosis and Alzheimer’s disease (106). Conversely, there is evidence for anti-inflammatory roles of LPC too, however mainly from research into peripheral inflammation and macrophages (107, 108). It is therefore unclear at this point whether the LPS induced decrease in hypothalamic LPC could be protective against or rather detrimental for the LPS induced neuroinflammation. More research is necessary in this direction, especially regarding LPCs function in the brain.

For the lipid class PC, the LPS effect depended on previous ELS exposure. While LPS increased its levels in control animals this was not the case in mice previously exposed to ELS. Next to its pivotal role in maintaining memory and nerve signaling as a precursor to acetylcholine, numerous studies have reported anti-inflammatory activities for PC (109). For example, LPS-induced acute inflammation in multiple peripheral organs (lung, liver, kidney) was reduced in mice injected with PC (109). While this data was not brain specific, possibly, the absence of the LPS induced increase in hypothalamic PC in ELS exposed mice is a sign of an increased pro-inflammatory state in LPS injected ELS mice as compared to CTL mice. Future studies are needed to understand exactly if and how brain PCs contribute to the ELS-induced effect on neuroinflammation.

Both ELS and early dietary ω6/ω3 ratio impacted the LPS induced lipid changes concerning TAG metabolism. In particular in CTL mice fed the high ω6/ω3 diet LPS increased three TAG species while in CTL mice fed the low ω6/ω3 diet LPS decreased 21 different TAG species. Increased TAG synthesis has been associated with increased inflammatory functions of peripheral macrophages in response to LPS (110, 111), whether this is also true for brain macrophages, microglia, remains to be investigated. Since we have previously reported effects of the ELS and the diet on microglia (21, 28), for future studies it would be interesting to investigate whether the early diet could affect microglia inflammatory signaling *via* altering TAG synthesis. There have been reports on the key role of dietary fatty acids and lipid metabolism in controlling microglial functionality (112, 113).

Considering the LPS induced reduction in ceramide, C16 and/or C18 ceramide were decreased in all LPS injected experimental groups when compared to their respective saline injected controls, except in ELS mice fed the high ω6/ω3 diet. This suggests that such reduction might be important for an appropriate response to LPS. We and others have shown previously that ELS leads to an exaggerated (neuro) inflammatory response to various secondary challenges such as amyloid accumulation (21), western style diet (114) and LPS (115). A lack of reduction in CERs for ELS under the high ω6/ω3 diet suggests that this might contribute to an altered neuroinflammatory response in the context of a second hit. Notably, in ELS mice fed the low ω6/ω3 diet this capacity was restored, thus potentially contributing to the beneficial and anti-inflammatory capacity of the diet. Whether this is indeed the case will need to be further elucidated. Considering the various functions of ceramides depending on their chain-length and the tissue (116), it remains unclear at this point what the functional implications are of the LPS induced reduction in hypothalamic ceramides.

Concerning oxylipins, consistent over all samples and experimental conditions and in line with previous studies, we detected an LPS induced increase in prostaglandin E_2_ (PGE_2_) (117). Notably, levels of hypothalamic PGE_2_ correlated with several pro-inflammatory plasma cytokines. PGE_2_ is an eicosanoid that plays an important role in acute and chronic inflammatory diseases (118–120). The increase in PGE_2_ in mice exposed to LPS is therefore a good marker for the induced inflammatory state in all experimental groups. Apart from the common increase in PGE_2_, the majority of the LPS induced changes were distinct for CTL and ELS exposed mice and unique for mice fed either the high or low ω6/ω3 diet. If and how the specific alterations contribute to the differential (neuro)immune response to LPS remains to be further elucidated.

There have been no other studies investigating the effects of LPS on brain oxylipins in interaction with previous exposure to both ELS and early dietary PUFA’s. Nevertheless, the direct effects of dietary PUFA’s on LPS induced brain oxylipins has been investigated. For example, Rey and colleagues reported that an ω3 LCPUFA dietary supplementation increased hippocampal ω3 oxylipins while decreasing ω6 oxylipins in response to LPS, as compared to mice fed a diet deficient in ω3 LCPUFAs (63). There are several differences between this and our study: the type and length of the dietary intervention, the LPS dose, the moment of lipid measurements and the studied brain region. Rey and colleagues used a long dietary intervention (2-month, ω3 supplemented versus ω3 deficient diet) directly followed by a low dose LPS injection (125 µg/kg i.p.) and measurements of hippocampal oxylipin levels. We used an early dietary intervention from P2–P42 and stimulation by a higher dose of LPS (5 mg/kg) and analysis of hypothalamic lipids only in adulthood. While we did not detect a low ω6/ω3 diet mediated increase in ω3 oxylipins in response to LPS, specifically in mice fed the low ω6/ω3 diet a reduction was observed in several ω6 AA derived oxylipins in response to LPS. The exact reasons why the early ω6/ω3 diet mostly affects mostly ω6 derived oxylipins remain to be understood. Nevertheless, our data suggests that even a relatively short early dietary intervention can have long-lasting anti-inflammatory actions upon an inflammatory challenge, thereby possibly promoting the resolution of inflammation.

In summary, LPS induced several changes in lipid species and oxylipins of which the increase in PGE_2_ is a clear indicator for the induced inflammatory state. The majority of the LPS induced alterations were however distinct for CTL and ELS exposed mice, and unique for mice fed either the high or low ω6/ω3 diet. This data suggests long-term programming by both ELS and the early diet, modulating lipid signaling in response to a later-life inflammatory challenge.

### 4.5 Limitation of our study

While our study presents some unique strength, as the experimental design and the unique combination of ELS and early-diet and immune challenge later in life, it also presents some limitations. Firstly, our study lacks the inclusion of female mice. As mentioned above, the current study is the follow up of our investigation on the protective effects of early dietary FAs against the ELS-induced cognitive and brain plasticity deficits in male mice (28). This original study included males only, because we had shown previously that the herein used ELS model affects neurogenesis and cognitive functions particularly in males (66). Nevertheless, studying if and how such effects are sex-dependent is an important future avenue due to the emerging evidence for sex differences in the response to ELS (121–124) and (early-life) dietary interventions (125, 126). In the current study we analyzed the lipid and oxylipin profiles in the hypothalamus, however it will be important in future studies to address also other brain regions that contribute to the ELS-induced phenotype. This will further our understanding on whether the here observed effects are common to the whole brain or rather brain-region specific and how the lipid profiles relate to the specific behavioral outcomes. Lastly, despite the fact that a large number of species was tested we chose not to correct for multiple testing as also clearly stated in the method section. We chose this analytical strategy due to two main reasons. Firslty, the current standard FDR correction is effective for independent data, but for dependent/corrected data such as metabolomics or lipidomics, it has been proven to be very conservative leading to the exclusion of true positives (127). Secondly, brain lipidomics is a relatively new field, especially in the context of early-life environmental factors such as stress and diet, which makes our study novel but also exploratory and hypothesis generating. We realize that as a result of our choice, few of the detected differences may have been due to chance, nonetheless we trust that many of them are likely meaningful biological differences as shown for example by the fact that we reproduce the expected LPS-induced changes in PGE2 as well as the ones highlighted in our discussion.

## 4.6 Conclusions

In conclusion, we show that ELS and early dietary ω6/ω3 PUFA ratio affect the hypothalamic lipid and oxylipin profile long-term, both under basal conditions and in response to an inflammatory challenge. Future studies are needed to elucidate the exact mechanisms leading to such long-term alterations in brain lipid and oxylipins and their functional implications. Nevertheless, these data give novel insights into how brain lipid profiles are part of the underlying mechanisms by which ELS exerts its effects on the brain and how the low ω6/ω3 diet might mitigate ELS-induced deficits, likely by modulating neuroinflammatory signaling as well as neuronal plasticity. Considering the fact that ELS and reduced intake of dietary PUFAs are risk factors for several diseases characterized by brain lipid imbalance, our work suggests that such lipid dysregulation might have an early-life origin, and that the observed alterations might contribute to the increased risk for these diseases.

## Data availability statement

The original contributions presented in the study are included in the article/**Supplementary Material**. Further inquiries can be directed to the corresponding author.

## Ethics statement

The animal study was reviewed and approved by the animal welfare body fo the University of Amsterdam and all experimental procedures were conducted under national law and European Union directives on animal experiments.

## Author contributions

KR and MA conceptualized the study and performed mouse-related experimental work. KR analyzed the data, prepared the figures and wrote the manuscript. JB performed experimental work and together with CC contributed to data analysis. AK conceptualized and supervised this study and reviewed and edited the manuscript. All authors contributed to editing of the manuscript and approved the submitted version.

## Funding

AK is supported by NWO Food Cognition and Behavior and Alzheimer Nederland, MG is supported by NWO XOmics project #184.034.019. GK is supported by the Dutch Research Council (NWO Vidi grant 91719305).

## Acknowledgments

We thank Niek Blomberg and Marieke Heijink (LUMC) for their expert assistance with lipid analysis.

## Conflict of interest

The authors declare that the research was conducted in the absence of any commercial or financial relationships that could be construed as a potential conflict of interest.

## Publisher’s note

All claims expressed in this article are solely those of the authors and do not necessarily represent those of their affiliated organizations, or those of the publisher, the editors and the reviewers. Any product that may be evaluated in this article, or claim that may be made by its manufacturer, is not guaranteed or endorsed by the publisher.

## Supplementary material

Supplementary Figure 1 Bodyweight, food intake, plasma corticosterone parameters. **(A)** ELS reduces bodyweight gain (two-way ANOVA: main effect condition p<0.05). **(B)** No significant effect of parameters on food intake. **(C)** Bodyweight is not different between experimental groups in adulthood. **(D)** 24 hours after LPS treatment, LPS and ELS decreased BW, but depended on condition and diet (three-way ANOVA: * = main effect condition (F1,40 = 8.049, p=0.007), ^: Main effect treatment (F1,68 = 13.191, p=0.001), &: interaction effect condition*treatment (F1,68 = 6.880, p=0.011), $: interaction effect condition*diet*treatment (F1,75 = 4.449, p=0.038) **(E)** LPS increased plasma CORT 24 hours after treatment, without further modulation by condition or diet. Three-way ANOVA: ^: main effect treatment (F75 = 414.5, p<0.001).

Supplementary Figure 2 Correlation plots between oxylipins/PUFAs per experimental group. **(A)** CTL-HRD-SAL, **(B)** ELS-HRD-SAL, **(C)** CTL-LRD-SAL, **(D)** CTL-HRD-LPS, **(E)** ELS-HRD-LPS, **(F)** CTL-LRD-LPS, **(G)** ELS-LRD-LPS. Abbreviations: CTL: control, ELS: early-life stress, SAL: saline, LPS: lipopolysaccharide, HRD: high ω6/ω3 ratio diet, LRD: low ω6/ω3 ratio diet

Supplementary material 1 Translation scheme of original Lipidyzer nomenclature to LIPID MAPS shorthand notation system

Supplementary Table 1 Descriptive statistics of plasma cytokines for the eight experimental groups. SEM: standard error of the mean, N: sample size.

Supplementary Table 2 Detected lipid classes and species

Supplementary Table 3 Raw values for broad lipidomic dataset per experimental group.

Supplementary Table 4 Raw values for oxylipin and upstream PUFA dataset per experimental group.

## Glossary

**Table:**

CTL	control
ELS	early-life stress
HRD	high ω6/ω3 ratio diet
LRD	low ω6/ω3 ratio diet
SAL	saline
LPS	lipopolysaccharide
HPLC-MS/MS	High performance liquid chromatography-tandem mass spectrometry
FA	fatty acids
PUFA	polyunsaturated fatty acids
P	postnatal day
DAG	diacylglycerol
TAG	triacylglycerol
FFA	free fatty acids
LPC	lysophosphatidylcholine
LPE	lysophosphatidylethanolamine
PC	phosphatidylcholine
PE	phosphatidylethanolamine
CER	ceramides
HCER	hexosylceramides
CE	cholesterol esters
SM	sphingomyelin
ALA	α-linolenic acid
EPA	Eicosapentaenoic acid
DHA	Docosahexaenoic acid
DPAn-3	Docosapentaenoic acid
n3	9-HoTrE: 9-hydroxy-octadecatrienoic acid
18-HEPE	18-hydroxy-eicosapentaenoic acid
4-HDHA	4-hydroxy-docosahexaenoic acid
7-HDHA	7-hydroxy-docosahexaenoic acid
10-HDHA	10-hydroxy-docosahexaenoic acid
14(S)-HDHA	14S-hydroxy-docosahexaenoic acid
17-HDHA	17-hydroxy-docosahexaenoic acid
19(20)EpDPA	19,20-epoxy-docosapentaenoic acid
LA	Linoleic acid
GLA	γ-linolenic acid
AA	Arachidonic acid
DPAn-6	Docosapentaenoic acid n6
DGLA	Dihomo-γ-linolenic acid
AdA	Adrenic acid
9-HoDE	9-Hydroxyoctadeca-dienoic acid
13-HoDE	13-hydroxy-octadecadienoic acid
8(9)EET	8;9-epoxy-eicosatrienoic acid, 11(12)EET
14(15)EET	14(15)-epoxy-eicosatrienoic acid
5-HETE	5-hydroxy-eicosatetraenoic acid
8-HETE	8-hydroxy-eicosatetraenoic acid
11-HETE	11-hydroxy-eicosatetraenoic acid
12-HETE	12S-hydroxy-eicosatetraenoic acid
15-HETE	15-hydroxy-eicosatetraenoic acid
17-OH-DH-HETE	17-OH-DH- hydroxyeicosatetraenoic acid
5-KETE	5-oxo-eicosatetraenoic acid
12-KETE	12-oxo-eicosatetraenoic acid
15-KETE	15-oxo-eicosatetraenoic acid
PGD_2_	Prostaglandin D2
PGE_2_	Prostaglandin E2
PGF_2α_	Prostaglandin F2a
8-iso-PGF2a	9α;11α,15S-trihydroxy-(8β)-prosta-5Z
PGJ_2_	Prostaglandin J2
15Deoxy PGJ_2_	15-deoxy-prostaglandin J2
TxB2	Thromboxane B2
