# Supplementary figures and images for: Early-life stress and dietary fatty acids impact the brain lipid/oxylipin profile into adulthood, basally and in response to LPS
Source: Front Immunol. 2022 Sep 5;13:967437. doi: 10.3389/fimmu.2022.967437 (PMC9484596; doi:10.3389/fimmu.2022.967437)

A)

Bodyweight gain P2 - P9

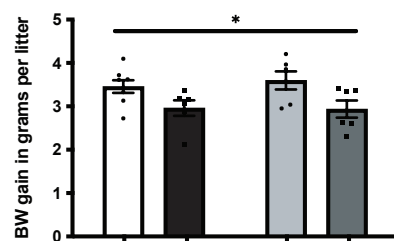

B)

Food intake dams P2 - P9

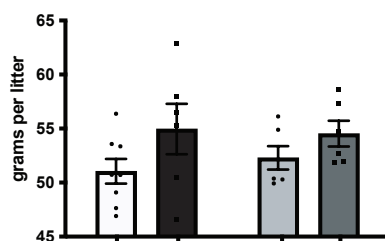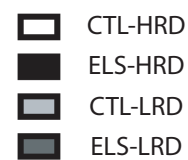

C)

BW before LPS

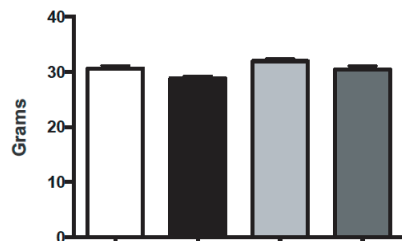

D)

BW after LPS

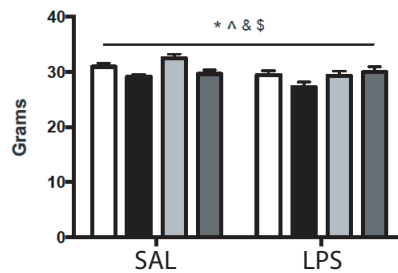

E)

CORT after LPS

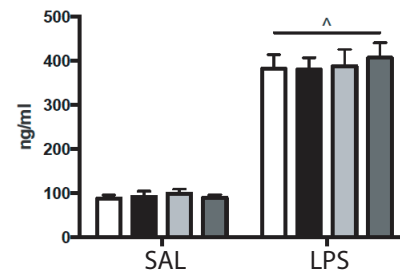

Supplement: Supplementary Figure 1 — Bodyweight, food intake, plasma corticosterone parameters. (A) ELS reduces bodyweight gain (two-way ANOVA: main effect condition p<0.05). (B) No significant effect of parameters on food intake. (C) Bodyweight is not different between experimental groups in adulthood. (D) 24 hours after LPS treatment, LPS and ELS decreased BW, but depended on condition and diet (three-way ANOVA: * = main effect condition (F1,40 = 8.049, p=0.007), ^: Main effect treatment (F1,68 = 13.191, p=0.001), &: interaction effect condition*treatment (F1,68 = 6.880, p=0.011), $: interaction effect condition*diet*treatment (F1,75 = 4.449, p=0.038) (E) LPS increased plasma CORT 24 hours after treatment, without further modulation by condition or diet. Three-way ANOVA: ^: main effect treatment (F75 = 414.5, p<0.001). [file DataSheet_1.pdf]
