# Supplementary material for: Early-life stress and dietary fatty acids impact the brain lipid/oxylipin profile into adulthood, basally and in response to LPS
Source: Front Immunol. 2022 Sep 5;13:967437. doi: 10.3389/fimmu.2022.967437 (PMC9484596; doi:10.3389/fimmu.2022.967437)
Supplement: Supplementary Figure 2 — Correlation plots between oxylipins/PUFAs per experimental group. (A) CTL-HRD-SAL, (B) ELS-HRD-SAL, (C) CTL-LRD-SAL, (D) CTL-HRD-LPS, (E) ELS-HRD-LPS, (F) CTL-LRD-LPS, (G) ELS-LRD-LPS. Abbreviations: CTL: control, ELS: early-life stress, SAL: saline, LPS: lipopolysaccharide, HRD: high ω6/ω3 ratio diet, LRD: low ω6/ω3 ratio diet [file DataSheet_2.pdf]

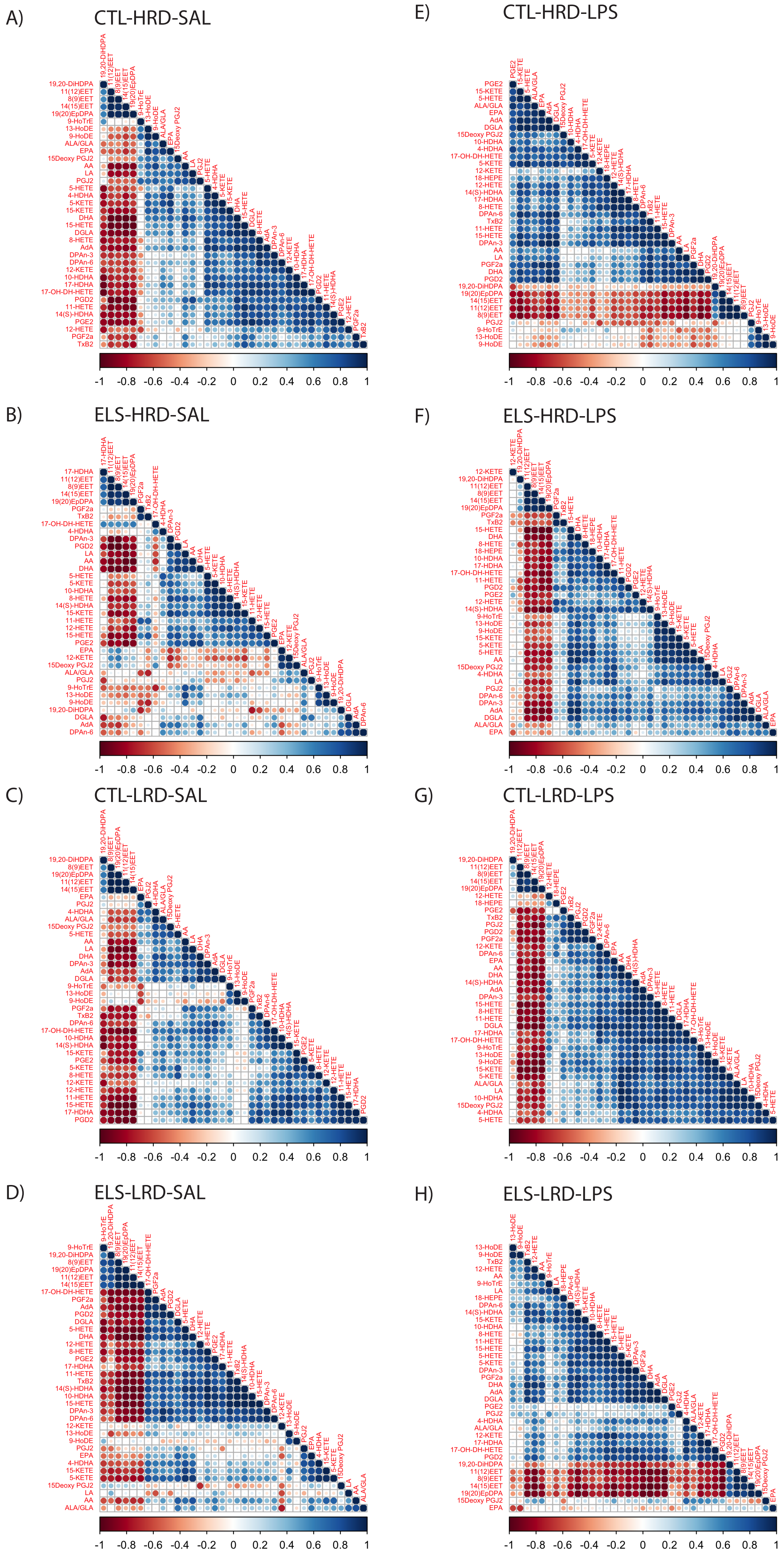
A)
CTL-HRD-SAL
E)
CTL-HRD-LPS
B)
ELS-HRD-SAL
F)
ELS-HRD-LPS
C)
CTL-LRD-SAL
G)
CTL-LRD-LPS
D)
ELS-LRD-SAL
H)
ELS-LRD-LPS
-1 -0.8 -0.6 -0.4 -0.2 0 0.2 0.4 0.6 0.8 1
