# Supplementary material for: Early-life stress and dietary fatty acids impact the brain lipid/oxylipin profile into adulthood, basally and in response to LPS
Source: Front Immunol. 2022 Sep 5;13:967437. doi: 10.3389/fimmu.2022.967437 (PMC9484596; doi:10.3389/fimmu.2022.967437)
Supplement: Supplementary material 1 — Translation scheme of original Lipidyzer nomenclature to LIPID MAPS shorthand notation system [file DataSheet_3.docx]

# Translation of Lipidyzer annotations to LIPIDMAPS shorthand annotation

| Lipidyzer abbreviation (examples) | LipidMaps shorthand annotation according to ^1,2^ |
| --- | --- |
| CE(14:0) | CE 14:0 |
| CER(18:0) | Cer d18:1/18:0 |
| DAG(12:0/16:0) | DG 12:0_16:0 |
| DCER(16:0) | Cer d18:0/16:0 |
| FFA(12:0) | FA 12:0 |
| HCER(16:0) | HexCer d18:1/16:0 |
| LCER(16:0) | LacCer d18:1/16:0 |
| LPC(16:0) | LPC 16:0 |
| LPE(16:0) | LPE 16:0 |
| PC(16:0/18:0) | PC 16:0_18:0 |
| PE(16:0/18:0) | PE 16:0_18:0 |
| PE(O-16:0/18:0) | PE O-16:0/18:0 |
| PE(P-16:0/18:0) | PE P-16:0/18:0 |
| SM(14:0) | SM d18:1/14:0 |
| TAG36:0-FA12:0 | TG 36:0-FA 12:0 |

^1^Liebisch, G. *et al.* Shorthand notation for lipid structures derived from mass spectrometry. *Journal of lipid research* **54**, 1523-1530 (2013).

^2^Liebisch, G. *et al.* Update on LIPID MAPS classification, nomenclature, and shorthand notation for MS-derived lipid structures. *Journal of lipid research* **61**, 1539-1555 (2020)
