# Supplementary material for: Early-life stress and dietary fatty acids impact the brain lipid/oxylipin profile into adulthood, basally and in response to LPS
Source: Front Immunol. 2022 Sep 5;13:967437. doi: 10.3389/fimmu.2022.967437 (PMC9484596; doi:10.3389/fimmu.2022.967437)
Supplement: Supplementary Table 2 — Detected lipid classes and species [file Table_2.docx]

**Supplementary table S2. Detected lipid classes and species**

| **Lipid class** | **Species** |
| --- | --- |
| **CE** | CE(14:0), CE(15:0), CE(16:0), CE(16:1), CE(17:0), CE(18:0), CE(18:1), CE(18:2), CE(18:3), CE(20:3), CE(20:4), CE(20:5), CE(22:5), CE(22:6) |
| **CER** | CER(16:0), CER(18:0), CER(24:0) |
| **DAG** | DAG(16:0/18:1), DAG(16:0/18:2), DAG(16:0/20:3), DAG(16:0/20:5), DAG(16:1/18:1), DAG(16:1/20:4), DAG(18:0/18:1), DAG(18:0/18:2), DAG(18:1/18:1), DAG(18:1/18:2) |
| **FFA** | FFA(12:0), FFA(18:1), FFA(18:2), FFA(18:3), FFA(20:3) |
| **HCER** | HCER(16:0), HCER(20:0), HCER(22:0), HCER(24:1) |
| **LPC** | LPC(16:0), LPC(18:1) |
| **LPE** | LPE(18:0) |
| **PC** | PC(16:0/18:1), PC(16:0/18:2), PC(16:0/20:4), PC(18:0/18:2), PC(18:1/18:1) |
| **PE** | PE(18:0/18:2), PE(16:0/20:1), PE(16:0/18:2) |
| **SM** | SM(18:0), SM(18:1), SM(24:1) |
| **TAG** | TAG42:0-FA16:0, TAG44:0-FA14:0, TAG44:0-FA16:0, TAG44:1-FA14:0, TAG44:1-FA16:0, TAG44:1-FA16:1, TAG44:1-FA18:1, TAG45:0-FA14:0, TAG46:0-FA14:0, TAG46:0-FA16:0, TAG46:1-FA14:0, TAG46:1-FA14:1, TAG46:1-FA16:0, TAG46:1-FA16:1, TAG46:1-FA18:1, TAG46:2-FA14:0, TAG46:2-FA14:1, TAG46:2-FA16:0, TAG46:2-FA16:1, TAG47:0-FA15:0, TAG47:0-FA16:0, TAG47:1-FA15:0, TAG47:1-FA18:1, TAG48:0-FA14:0, TAG48:0-FA16:0, TAG48:0-FA18:0, TAG48:1-FA14:0, TAG48:1-FA16:0, TAG48:1-FA16:1, TAG48:1-FA18:1, TAG48:2-FA14:0, TAG48:2-FA14:1, TAG48:2-FA16:0, TAG48:2-FA16:1, TAG48:2-FA18:1, TAG48:2-FA18:2, TAG48:3-FA14:1, TAG48:3-FA16:1, TAG48:3-FA18:2, TAG48:4-FA16:1, TAG48:4-FA18:2, TAG49:0-FA15:0, TAG49:0-FA16:0, TAG49:0-FA17:0, TAG49:1-FA15:0, TAG49:1-FA16:0, TAG49:1-FA17:0, TAG49:1-FA18:1, TAG49:2-FA15:0, TAG49:2-FA16:0, TAG49:2-FA18:1, TAG49:3-FA16:1, TAG50:0-FA16:0, TAG50:0-FA18:0, TAG50:1-FA14:0, TAG50:1-FA16:0, TAG50:1-FA16:1, TAG50:1-FA18:0, TAG50:1-FA18:1, TAG50:2-FA14:0, TAG50:2-FA16:0, TAG50:2-FA16:1, TAG50:2-FA18:0, TAG50:2-FA18:1, TAG50:2-FA18:2, TAG50:3-FA14:0, TAG50:3-FA14:1, TAG50:3-FA16:0, TAG50:3-FA16:1, TAG50:3-FA18:1, TAG50:3-FA18:2, TAG50:4-FA16:1, TAG50:4-FA18:2, TAG51:0-FA16:0, TAG51:0-FA17:0, TAG51:0-FA18:0, TAG51:1-FA16:0, TAG51:1-FA17:0, TAG51:1-FA18:0, TAG51:1-FA18:1, TAG51:2-FA15:0, TAG51:2-FA16:0, TAG51:2-FA16:1, TAG51:2-FA17:0, TAG51:2-FA18:1, TAG51:3-FA16:1, TAG51:3-FA18:2, TAG51:4-FA16:1, TAG51:4-FA18:2, TAG52:0-FA16:0, TAG52:0-FA18:0, TAG52:1-FA16:0, TAG52:1-FA16:1, TAG52:1-FA18:0, TAG52:1-FA18:1, TAG52:1-FA20:1, TAG52:2-FA16:0, TAG52:2-FA16:1, TAG52:2-FA18:0, TAG52:2-FA18:1, TAG52:2-FA18:2, TAG52:2-FA20:2, TAG52:3-FA16:0, TAG52:3-FA16:1, TAG52:3-FA18:0, TAG52:3-FA18:1, TAG52:3-FA18:2, TAG52:3-FA20:3, TAG52:4-FA16:0, TAG52:4-FA16:1, TAG52:4-FA18:1, TAG52:4-FA18:2, TAG52:4-FA18:3, TAG52:4-FA20:3, TAG52:4-FA20:4, TAG52:5-FA16:0, TAG52:5-FA16:1, TAG52:5-FA18:1, TAG52:5-FA18:2, TAG52:5-FA18:3, TAG52:5-FA20:5, TAG52:6-FA22:6, TAG52:7-FA16:0, TAG53:1-FA16:0, TAG53:1-FA18:1, TAG53:2-FA16:0, TAG53:2-FA17:0, TAG53:2-FA18:1, TAG53:3-FA16:0, TAG53:3-FA18:2, TAG53:4-FA16:0, TAG53:4-FA18:2, TAG53:5-FA20:4, TAG54:0-FA18:0, TAG54:1-FA16:0, TAG54:1-FA18:0, TAG54:1-FA18:1, TAG54:2-FA16:0, TAG54:2-FA18:0, TAG54:2-FA18:1, TAG54:2-FA18:2, TAG54:3-FA16:0, TAG54:3-FA16:1, TAG54:3-FA18:0, TAG54:3-FA18:1, TAG54:3-FA18:2, TAG54:3-FA20:2, TAG54:3-FA20:3, TAG54:4-FA16:0, TAG54:4-FA16:1, TAG54:4-FA18:0, TAG54:4-FA18:1, TAG54:4-FA18:2, TAG54:4-FA20:3, TAG54:4-FA20:4, TAG54:4-FA22:4, TAG54:5-FA16:0, TAG54:5-FA18:1, TAG54:5-FA18:2, TAG54:5-FA18:3, TAG54:5-FA20:3, TAG54:5-FA20:4, TAG54:5-FA22:4, TAG54:5-FA22:5, TAG54:6-FA22:5, TAG54:6-FA22:6, TAG55:2-FA18:1, TAG55:3-FA18:1, TAG55:4-FA18:1, TAG55:5-FA18:1, TAG56:1-FA18:1, TAG56:2-FA16:0, TAG56:3-FA18:0, TAG56:3-FA18:1, TAG56:4-FA16:0, TAG56:4-FA18:0, TAG56:4-FA18:1, TAG56:4-FA18:2, TAG56:4-FA20:2, TAG56:4-FA20:3, TAG56:4-FA22:4, TAG56:5-FA16:0, TAG56:5-FA18:0, TAG56:5-FA18:2, TAG56:5-FA20:3, TAG56:5-FA22:4, TAG56:5-FA22:5, TAG56:6-FA22:4, TAG56:6-FA22:5, TAG56:6-FA22:6, TAG58:3-FA18:1, TAG58:6-FA22:5 |
